# Challenging the Thrift Paradigm: When Decreasing Income Trajectory Drives Expensive Non-Conformity

**DOI:** 10.3390/bs16091648

**Published:** 2026-09-14

**Authors:** Zhengnan He, Mingqian Li, Jaimie W. Lien, Yuetong Lu

**Affiliations:** 1 Center for Economic Research, Shandong University, Jinan 250100, China; zhengnan.academic@gmail.com; 2 Zaozhuang Tobacco Monopoly Bureau, Zaozhuang 277000, China; lyt3511964179@163.com

**Keywords:** income anticipation, social information, counter-conformity, consumption behavior, cheap-salience

## Abstract

In an increasingly complex modern social information environment, in which consumers are exposed to both changes in economic conditions and pervasive social comparison cues, there remains limited research exploring the relationship between financial anticipation, social signals, and consumption choices. Using an experimental approach, we examine how income trajectories and social information shape consumption choices. Participants in our online decision task were randomly exposed to information indicating either increasing or decreasing income trajectories, alongside social information about the popularity of a relatively cheap or expensive choice of a specific type of product, among individuals with the same income trajectory. Firstly, we find that cheap information during economic decline undermines the standard prediction that anticipated income decline leads consumers to prefer cheaper products. Specifically, the results suggest a significant non-conformity effect specific to the scenario of anticipated income decline: when income was expected to decrease and the social information indicated a majority choice of cheap products, participants were more likely to select the expensive products. On the other hand, social information had no significant impacts when income was expected to increase. Our findings challenge the conventional intuition that social information indicating a cheap majority encourages frugal consumption, instead suggesting a counter-conformity pattern under anticipated income decline.

## 1. Introduction

In modern economic systems, individual decision-making is often constrained by dual frictions: aggregate fluctuations in the economic environment and peer effects within the social information landscape. As global economic uncertainty becomes normalized, consumers face not only idiosyncratic shocks to future income streams but also reside within a highly transparent network of social comparison. This has heightened the salience of income trajectories and social comparison in everyday consumption choices. These forces raise a fundamental question: how do consumers integrate anticipations about their future income with social information when choosing between cheap and expensive products?

Standard consumption theory predicts that anticipated income declines should strengthen preferences for cheaper goods through precautionary saving and tighter budget constraints (Friedman, 1957). At the same time, a large body of empirical evidence documents that social information (e.g., what others choose) systematically shapes individual behavior (Banerjee, 1992). However, how these two forces interact remains insufficiently understood, particularly in environments characterized by economic pressures and different income trajectories.

This study employs a randomized controlled experiment to identify the mechanisms of consumption choice under economic pressure by exogenously manipulating income trajectories and social information. Within a hypothetical context of economic downturn and employment instability, participants were exposed to social information derived from pilot participants’ choices between cheaper and more expensive products. Within the social information conditions, the same underlying pilot choice distribution was presented by displaying either the proportion choosing the cheaper option or the complementary proportion choosing the more expensive option. The study’s core finding indicates a counter-intuitive “non-conformity effect”: in the treatment group under income decline, when the social information was “cheap-preferring,” the probability of participants selecting expensive products significantly increased. This suggests that during economic downturns, social information about cheaper choices may in fact fail to induce frugality and instead potentially act as a psychological threat, triggering consumers to engage in counter-conformity via expensive purchases as a possible channel for avoiding social status devaluation.

A large literature in microeconomics and behavioral economics studies how information affects consumption choices. Early work emphasized expectations about lifetime income as a key determinant of consumption smoothing (Friedman, 1957). More recent studies highlight the role of economic uncertainty and pessimistic income trajectories in shaping precautionary behavior (Coibion et al., 2022; Guvenen et al., 2021). These studies consistently predict reduced demand for expensive goods when income prospects deteriorate.

Another strand of literature documents that economically equivalent information can lead to systematically different choices depending on how information is framed (DellaVigna, 2009). In consumption contexts, price and reference points have been shown to influence demand, perceived value, and willingness to pay (Chetty et al., 2009). However, asset price expectations yield heterogeneous effects: while renters reduce spending when anticipating higher home prices due to housing cost burdens, homeowners’ spending remains unaffected, reflecting wealth offsetting (Chopra et al., 2025). This highlights how economic constraints and identity-based motivations can interact with information to shape consumption decisions.

A growing literature emphasizes the influence of social information and peer effects on economic behavior (Banerjee, 1992). Information about majority choices often induces conformity, especially under uncertainty (Beshears et al., 2008). However, recent work also suggests that social signals can also generate a deviation from the conformity when status or identity concerns are salient (Bursztyn et al., 2020; Grossman & Helpman, 2021). Under situations of increasing or decreasing income trajectories, an individual’s income trend or economic condition can serve as an identity factor that influences conformity and a deviation from the conformity behavior. Our experiment enables a systematic examination of the impacts of the observable choices of others on an individual’s own consumption choice under a shared income trajectory.

This issue is of particular importance in addressing questions of whether economic downturns inevitably lead to a perpetual spiral of ‘downgraded’ consumption, or whether there are fundamental counteracting forces at the microeconomic and psychological level, which can naturally mitigate such undesirable macroeconomic outcomes. The results in our study suggest that perhaps societies need not be overly concerned about endless cycles of downgraded consumption. The reason is that consumers may naturally gravitate towards a tendency to distinguish themselves from their peers in the same income trajectory group, by choosing more expensive products when their peers choose cheaper products. However, this finding also bears a cautionary message in terms of the consumer welfare of consumption choices, in that the tendency to gravitate towards more expensive options from a social status motive can exert additional economic pressures on consumers during periods of income decline.

The remainder of this paper proceeds as follows: Section 2 describes the conceptual framework and hypotheses. Section 3 introduces the detailed study design and implementation procedure. Section 4 presents the main results and robustness checks, along with heterogeneity analysis. Section 5 concludes and discusses policy implications.

## 2. Conceptual Framework and Hypotheses

Recent research emphasizes that consumption responses to income trajectories depend critically on perceived economic security rather than expected income levels alone. Empirical evidence indicates that reference dependence can shape dynamic decisions under uncertainty (Lien & Zheng, 2015). Evidence from high-frequency administrative and survey data shows that households exposed to negative income news sharply reduce discretionary spending, consistent with heightened consumption discipline rather than smooth adjustment of consumption (Ganong et al., 2020). Related work further documents that perceived labor market fragility amplifies conservative consumption behavior even in the absence of realized income losses (Baker et al., 2023). In this context, an anticipated income decline reduces the effective consumption constraints and increases sensitivity to price-related considerations; thus, consumers facing pessimistic income trajectories should be expected to display stronger preferences for cheaper options.

A growing literature indicates that social information functions as a significant behavioral influence when individuals face uncertainty and limited confidence in their private assessments. Empirical evidence indicates that households systematically update beliefs and economic plans in response to socially transmitted narratives, even when such narratives are only loosely connected to fundamentals (D’Acunto et al., 2022). In consumption contexts, information about majority choices can therefore serve as a coordination device, reducing perceived decision risk and cognitive effort. This role of social information is expected to intensify when economic prospects deteriorate. Anticipated income decline increases uncertainty about future resources, making individuals more reliant on external information such as majority behavior.

Descriptive social information can identify the majority choice while also conveying the distribution of choices across the available alternatives. The same underlying choice distribution can therefore be presented by highlighting different options. For example, if 60% of pilot participants chose the cheaper product, this distribution can be presented either as “60% chose the cheaper product” or, equivalently, as “40% chose the more expensive product.” We refer to these complementary information presentations as cheap salience and expensive salience, respectively. Cheap salience displays the proportion choosing the cheaper option, whereas expensive salience displays the complementary proportion choosing the more expensive option.

Although these two statements convey the same underlying choice distribution, emphasizing different product options may direct attention toward different aspects of the social information. More broadly, research on information presentation shows that logically equivalent descriptions can produce different evaluations when they emphasize different attributes of an object or decision (LeBoeuf & Shafir, 2006). Related research on descriptive norm communication likewise suggests that behavioral responses may depend on how norm-relevant behavior is expressed, although evidence of such presentation effects varies across contexts (Gerber et al., 2018). Applied to the present setting, highlighting the proportion choosing the cheaper product may increase the prominence of thrift and economic restraint, whereas highlighting the complementary proportion choosing the more expensive product may draw attention toward the premium alternative. This distinction should not be equated with Cialdini et al.’s concept of negative social proof. Negative social proof occurs when a message intended to discourage an undesirable behavior emphasizes that many people engage in that behavior, thereby inadvertently communicating that it is descriptively common (Cialdini et al., 2006). In our setting, neither the cheaper nor the more expensive choice was explicitly characterized as desirable, undesirable, appropriate, or inappropriate. Instead, the cheap-salience and expensive-salience conditions communicated complementary proportions from the same pilot choice distribution while directing attention toward different product options.

Economic pressure may simultaneously heighten sensitivity to social rank and identity concerns. Recent research shows that perceived downward mobility risk can trigger compensatory behavior aimed at preserving one’s social standing (Bonomi et al., 2021). Related evidence demonstrates that threats to self-image induce compensatory actions that are often costly in material terms (Mazar et al., 2008). Applied to consumption choices, when cheap options become socially associated with vulnerability or decline, individuals may deliberately deviate from the cheap-majority consumption pattern. In such contexts, choosing expensive products can function as a costly signal of resilience, self-worth or social distinction.

When an individual’s income is expected to increase, economic uncertainty is relatively low and perceived budget constraints are relaxed. Under such conditions, individuals are less dependent on external social cues and more likely to rely on stable preferences or intrinsic product valuation. D’Acunto et al. (2022) found support for this, which suggests that the behavioral influence of social information weakens when decision-makers feel economically secure. Therefore, the majority choice of whether it is cheap or expensive should not systematically affect consumption decisions when income prospects are favorable.

Two features of the social information should therefore be distinguished. First, majority status indicates whether the cheaper or the more expensive option was selected by more than half of the corresponding pilot participants. Second, information salience indicates whether participants were shown the proportion choosing the cheaper option or the complementary proportion choosing the more expensive option. Consequently, when the cheaper option constituted the pilot majority, cheap salience produced cheap-majority information, whereas expensive salience produced the complementary expensive-minority information. When the more expensive option constituted the pilot majority, the corresponding presentations were cheap-minority information and expensive-majority information. Majority status was derived from the pilot choices at the product level, whereas information salience was manipulated between participants. With these concepts and theories, we formulate the following hypotheses about the relationship between income trajectories, social information and the choices of cheaper products. Our information disclosure treatments and subsequent statistical analysis will shed light on whether there are differences in conformity behavior across income trajectories conditions and whether information about others’ choices of cheap salient products versus expensive salient products accounts for these patterns in meaningful ways:

Our first hypothesis addresses the general pattern of product purchase between anticipated income decrease versus increase.

**Hypothesis** **1.** *Decision-makers under anticipated income decline choose cheap products more frequently than those anticipating income increase.*

This hypothesis straightforwardly reflects the notion that consumers under expected income decrease gravitate towards cheaper products. The reasoning is that when income is expected to decline, individuals face heightened economic insecurity and increased concern over future resources. Standard economic reasoning predicts that such conditions strengthen consumption discipline and amplify price sensitivity. This income-driven preference for cheaper options should manifest as a baseline pattern, regardless of whether social information is available.

Our second hypothesis addresses product choices under income decline, specifically whether consumers tend to conform to majority choices or not. While Hypothesis 1 predicts a general shift toward cheaper products under income decline, social information may trigger more complex responses when frugal consumption is socially salient. Based on the literature on social information and influence, we expect social information to suggest conformity effects. Building on this foundation, Hypothesis 2 examines how social information affects cheap product consumption under anticipated income decline. However, at a more granular level, we recognize that two competing mechanisms may be at play (“A” denotes competing alternative directional hypotheses):    

**Hypothesis $2_{A1}$ (Conformity to Frugal Patterns). **  *Social information indicating that the majority of consumers facing the same anticipated income trajectory chose cheap products will reinforce frugal consumption patterns.*

This hypothesis posits that social information serve a dual function as both informational guidance and social justification, strengthening the normative pull toward prudent consumption and conferring legitimacy on frugal choices. Consequently, individuals will conform to the frugal majority.

Alternative mechanism (counter-conformity): Economic decline may threaten individuals’ perceived social status and self-image. When cheap consumption becomes socially salient through information about majority purchases, it may signal overall vulnerability or downward mobility among those facing anticipated income decline. In response, individuals may be motivated to specifically avoid conforming to the majority choice. One explanation is that information about majority cheap purchases triggers a costly counter-conformity motive, whereby individuals attempt to achieve social distinction and resist association with the economically constrained group.

**Hypothesis $2_{A2}$ (Social Information and Counter-Conformity). **  *Under anticipated income decline, participants exposed to social information indicating an underlying cheap majority will choose cheaper products less frequently than those receiving no information, thereby exhibiting a counter-conformity pattern.*

### 2.1. Social Information Moderates the Income-Conformity Relationship

While the previous hypotheses specify how the purchase of differently priced products depend on expected income trends (income decrease, specifically), and potentially on social information, our next set of hypotheses specifically addresses the relationship between conformity and income trajectories. We define conformity in this context as choosing the product that the majority of consumers chose, based on the social information provided to the decision-maker.

The present design allows us to examine observed conformity across income trajectories, although this comparison requires careful interpretation. Because the social information reflected the actual choices observed in the pilot sample, the majority status of the product options was not experimentally balanced across income-trajectory conditions. In particular, the cheaper option constituted the pilot majority for nine of the ten products under the income-decrease scenario, compared with four products under the income-increase scenario. Because participants assigned to the income-decrease scenario were also expected to have a stronger baseline preference for cheaper products, as proposed in Hypothesis 1, choosing the majority option was more closely aligned with their underlying product preferences. Consequently, a higher observed conformity rate under income decrease compared with income-increase condition could reflect responses to social information, underlying preferences, or a combination of the two.

**Hypothesis** **3.**  *Income-declining individuals exhibit higher conformity than those in income-increasing individuals (baseline, potentially confounded).*

Once cheap salience information is accounted for, the conformity gap may disappear if the observed difference in Hypothesis 3 is driven by preference rather than genuine social influence.

**Hypothesis** **4.**  *After accounting for information salience, no statistically detectable difference in observed conformity remains between the income-decrease and income-increase conditions.*

Separately, Hypotheses $4_{A1}$ and $4_{A2}$ address whether the option emphasized in the presentation of social information is associated with observed conformity. Because cheap salience displays the proportion choosing the cheaper option regardless of whether that option constitutes the majority or minority choice, these hypotheses concern information presentation rather than majority status. Emphasizing the cheaper option may increase conformity by directing attention toward economically restrained behavior, or it may reduce conformity by eliciting reactance or heightening concerns about social status.   

**Hypothesis $4_{A1}$. **  *Cheap salience is positively associated with observed conformity.*

**Hypothesis $4_{A2}$. **  *Cheap salience is negatively associated with observed conformity.*

### 2.2. Information Effects Under Income Increase

Hypothesis 5 examines how social information shapes product choice for income-increasing individuals. When income is anticipated to increase, economic uncertainty is relatively low and perceived budget constraints are relaxed, making individuals less reliant on external social cues. Hypothesis 5 therefore serves as a general null hypothesis against the no-information baseline.

**Hypothesis** **5.**  *When income is anticipated to increase, social information, whether cheap or expensive salience, has no systematic effect on product choices relative to the no-information baseline.*

Building on Hypothesis 5, Hypothesis 6 examines a more specific directional question: whether the *form* of information presentation within the social information conditions produces differential responses, independent of the overall null effect relative to no information.

**Hypothesis** **6.**  *Among individuals anticipating an income increase, exposure to expensive-minority information leads to more frequent cheap-product choices than exposure to the complementary cheap-majority information.*

## 3. Study Design

### 3.1. Experimental Design

We employed a 2×3 between-subjects design to examine how anticipated income trajectory and social consumption information relate to product choices. Participants were randomly assigned to either an income-increase or income-decrease scenario and to one of three information conditions: no information, cheap salience, or expensive salience. The no-information condition served as the baseline, while the cheap-salience and expensive-salience conditions are collectively referred to social information conditions throughout the paper.

The combination of the two income-trajectory scenarios and three information conditions yielded six between-subjects experimental conditions. A total of 480 participants were recruited via the online platform Credamo and initially assigned randomly to one of the six conditions (n = 80 per condition), resulting in a final valid analytical sample of 468 participants.^1^ Before data collection, we prepared an analysis plan in the OSF registration system on 9 February 2025. However, the plan was inadvertently left in draft form and was not formally submitted. Nevertheless, the time-stamped pre-registration materials are available for transparency and reference.^2^

All participants completed the same 10 product-choice tasks. Within each income-trajectory scenario, the actual choices observed in the corresponding pilot sample determined whether the cheaper or the more expensive option constituted the majority choice for each product pair. Majority status was therefore information derived from actual pilot data rather than a separately randomized between-subjects factor. Consequently, the information presented in the cheap-salience condition could constitute either cheap-majority information, when more than half of the pilot participants selected the cheaper option, or cheap-minority information, when fewer than half selected it. Correspondingly, the complementary information presented in the expensive-salience condition constituted expensive-minority information or expensive-majority information, respectively.

Within the social information conditions, each underlying pilot choice distribution was presented in one of two complementary ways. Participants in the cheap-salience condition viewed the proportion of pilot participants who selected the cheaper option, whereas those in the expensive-salience condition viewed the complementary proportion who selected the more expensive option. Thus, information salience determined which side of the same underlying choice distribution was emphasized without changing the product’s majority–minority relationship. Accordingly, the experimental conditions varied whether social information was provided and, when provided, whether the proportion choosing the cheaper or the more expensive option was displayed. Our primary counter-conformity analyses focus on comparisons between the no-information and social information conditions under the hypothetical income-decrease scenario.

### 3.2. Social Information Construction

The pilot study was conducted on 10 February 2025, with a total of 60 participants, comprising 30 participants in each income-trajectory condition. We used the pilot participants’ actual choices to construct the social information stimuli. This decision was guided by the non-deception practice discussed in experimental economics, which avoids the provision of misleading information to subjects. A longstanding standard in experimental economics is that information presented to participants should not contain deceptions, as doing so may weaken their trust in the experimental instructions and the information on which their decisions are based, thereby complicating the interpretation of their subsequent choices (for discussions, see (Charness et al., 2022; Hertwig & Ortmann, 2012; Jamison et al., 2008)). We acknowledge that alternative experimental practices are common and acceptable across different fields of study and, furthermore, that using pilot data is not the only method of adhering to non-deception. For example, real consumption data from outside our experimental setting could be provided to subjects. Related studies have likewise used actual choices observed in prior or auxiliary samples as the basis for social-information interventions (Dimant & Gesche, 2023; Karapetian et al., 2025), while field research has used information reflecting prevailing behavioral norms in naturally occurring settings (Su et al., 2026). However, to maintain the internal comparability between the social information and subjects’ choices from the perspective of the subjects themselves, while also avoiding any manipulation of the actual choice proportions, we used the proportions generated by a pre-specified number of pilot participants’ actual choices under the same income-trajectory scenarios and product-choice tasks as the source of social consumption choice information. Treatments then varied based on the provision and presentation of this information, so that the experimental variation was not on the content of information itself, but rather in whether the information was provided and how it was presented.

In this study, the pilot served specifically to provide empirically grounded material for constructing the experimental stimuli, rather than as a basis for hypothesis testing or population-parameter estimation. We did not use the pilot data to test the research hypotheses, estimate treatment effects, or precisely estimate the population proportions choosing the cheaper or more expensive products. Accordingly, the pilot sample size was not determined by the statistical power requirements of confirmatory hypothesis testing, nor are the resulting proportions interpreted as precise estimates of population-level product preferences. As a descriptive consistency check, we compared the pilot proportions with the choices subsequently observed in the corresponding no-information conditions. The item-level patterns were strongly correlated across the 10 products under both anticipated income increase (r=0.964) and anticipated income decline (r=0.971), with mean absolute differences of 4.84 and 3.02 percentage points, respectively. These comparisons are reported in Appendix A Table A10. They imply that the pilot-generated choice patterns were broadly consistent with those subsequently observed in the absence of social information.

Using the actual choices observed in the pilot study for the same product-choice tasks, we first classified each product pair according to whether the cheaper or the more expensive option constituted the majority choice within the corresponding income-trajectory scenario.

Under the income-increase scenario, four product pairs had a cheap majority (Q3, Q4, Q8, and Q12), whereas six had an expensive majority (Q5, Q6, Q7, Q9, Q10, and Q11). Under the income-decrease scenario, nine product pairs had a cheap majority (Q3, Q4, Q5, Q6, Q7, Q8, Q10, Q11, and Q12), whereas only Q9 had an expensive majority. These majority classifications were therefore determined by the pilot choices and varied across products and income-trajectory scenarios.

Within the social information conditions, each underlying pilot choice distribution was presented in one of two mathematically complementary ways that conveyed the same underlying choice information:

Cheap-salience condition: Participants viewed the proportion of pilot participants who selected the cheaper option.

Expensive-salience condition: Participants viewed the complementary proportion of pilot participants who selected the more expensive option.

Thus, information salience determined which side of the same underlying choice distribution was displayed without altering the product’s majority–minority relationship. For a cheap-majority product, the cheap-salience condition presented cheap-majority information, whereas the expensive-salience condition presented the complementary expensive-minority information. For an expensive-majority product, the cheap-salience condition presented cheap-minority information, whereas the expensive-salience condition presented expensive-majority information.

**Figure 1:**
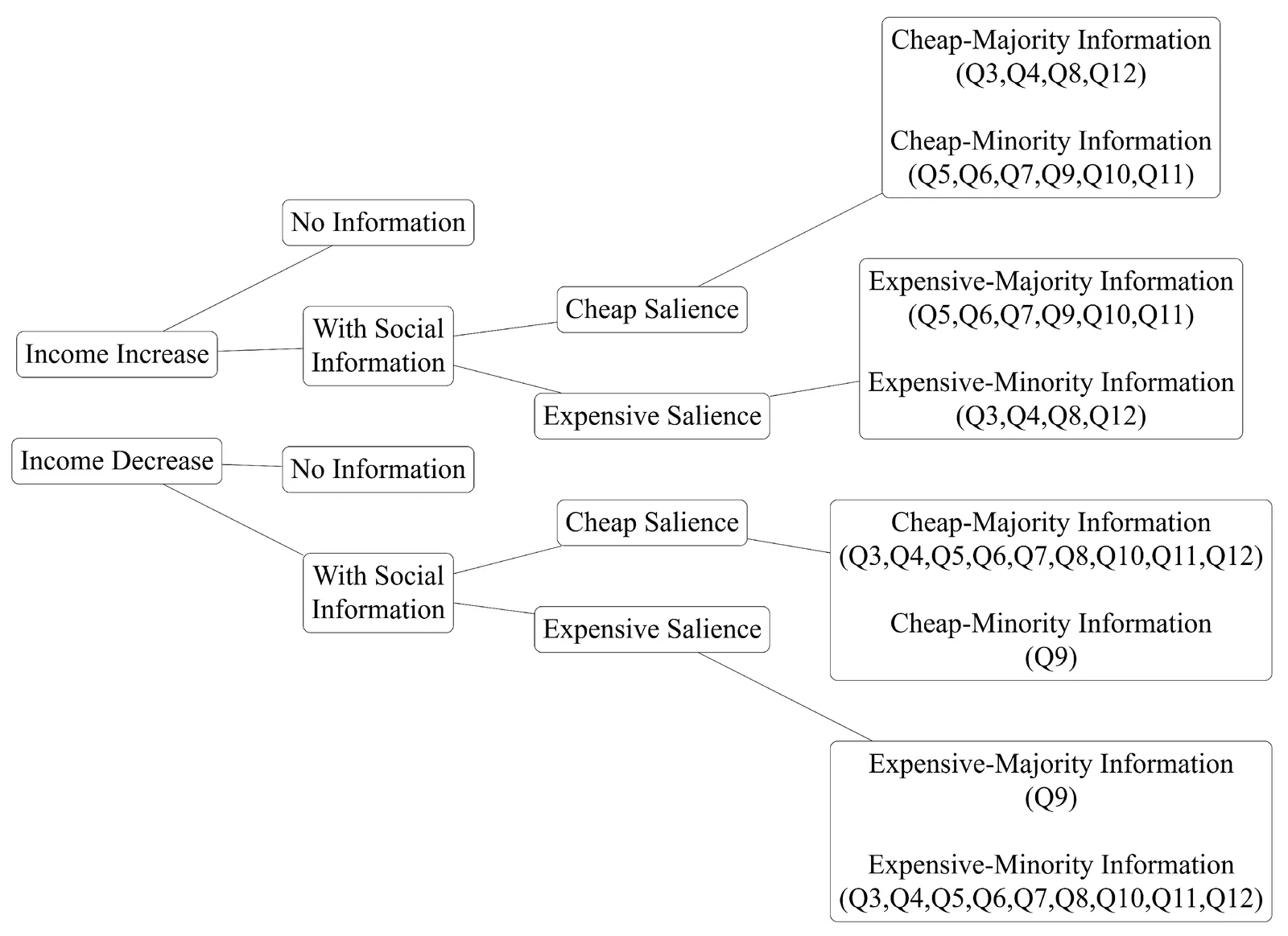
Treatment group chart. Note: The social information displayed in the treatments was derived from the actual choices of 60 pilot participants (30 in each income-trajectory condition). The cheap-salience condition displayed the proportion choosing the cheaper option, whereas the expensive-salience condition displayed the complementary proportion choosing the more expensive option. Accordingly, whether the displayed information represented a majority or minority varied across products and income-trajectory conditions, as determined by the underlying pilot choice data.

Participants in the no-information conditions completed the same product-choice tasks but received no information about the choices of pilot participants. These conditions served as the reference groups for estimating the effects of social information. Figure 1 summarizes the structure of the experimental conditions and the relationship between pilot-derived majority status and information presentation.

### 3.3. Experimental Procedure

The experimental procedure was conducted online using the platform Credamo. After providing informed consent and reading task instructions, participants were exposed to the income trajectory condition. To enhance psychological engagement with the scenario, participants were asked to imagine themselves in a specific economic situation. They read a detailed textual scenario describing either declining or rising income anticipations, accompanied by a simulated bar graph visualizing the income trajectory over time. In the income-decreasing condition, the scenario described economic downturn, industry-wide decline, and job instability as factors causing continuous income decline. In the income-increasing condition, participants read a parallel scenario depicting a positive economic outlook, industry growth, and stable career advancement leading to rising income. This imaginative scenario was designed to create stronger immersion, making the scenario more convincing.

To facilitate verification that participants thoroughly processed the scenario and actively engaged with the imagined situation, they were asked two engagement questions (Q1 and Q2) about the hypothetical scenario’s impacts on their lives. These questions encouraged participants to think realistically about the scenario and adopt the corresponding mentality, thus deepening engagement with the treatment. Following the scenario-engagement questions, participants completed an attention check that instructed them to select a specific option. Those who failed were automatically terminated from the survey task and replaced until 480 valid responses were collected; thus, all 480 participants passed the attention check.

Our study employs a scenario-based vignette design: a detailed written description of an economic situation accompanied by a visualized income trajectory graph. Vignette-based experiments have been widely validated as a method for inducing hypothetical economic states. Hainmueller et al. (2015) demonstrated that responses to carefully constructed vignettes closely predict real-world behavior, and Aguinis and Bradley (2014) provide methodological best-practice guidance confirming the validity of vignette designs for causal inference in behavioral research. To encourage and help verify active engagement with the intended income scenario, we included two post-scenario questions: participants rated their satisfaction with the imagined income trajectory (Q1; 9-point Likert scale) and identified its anticipated impacts on their lives (Q2; multiple choice). As expected, income-decrease participants reported significantly lower satisfaction than income-increase participants (M=2.56 vs. M=7.76, t(466)=39.97, p<0.001), and selected negative life impacts (e.g., reduced financial security, lower consumption) at significantly higher rates. These results confirm that the scenario successfully induced the intended differential mindset across conditions.

**Figure 2:**
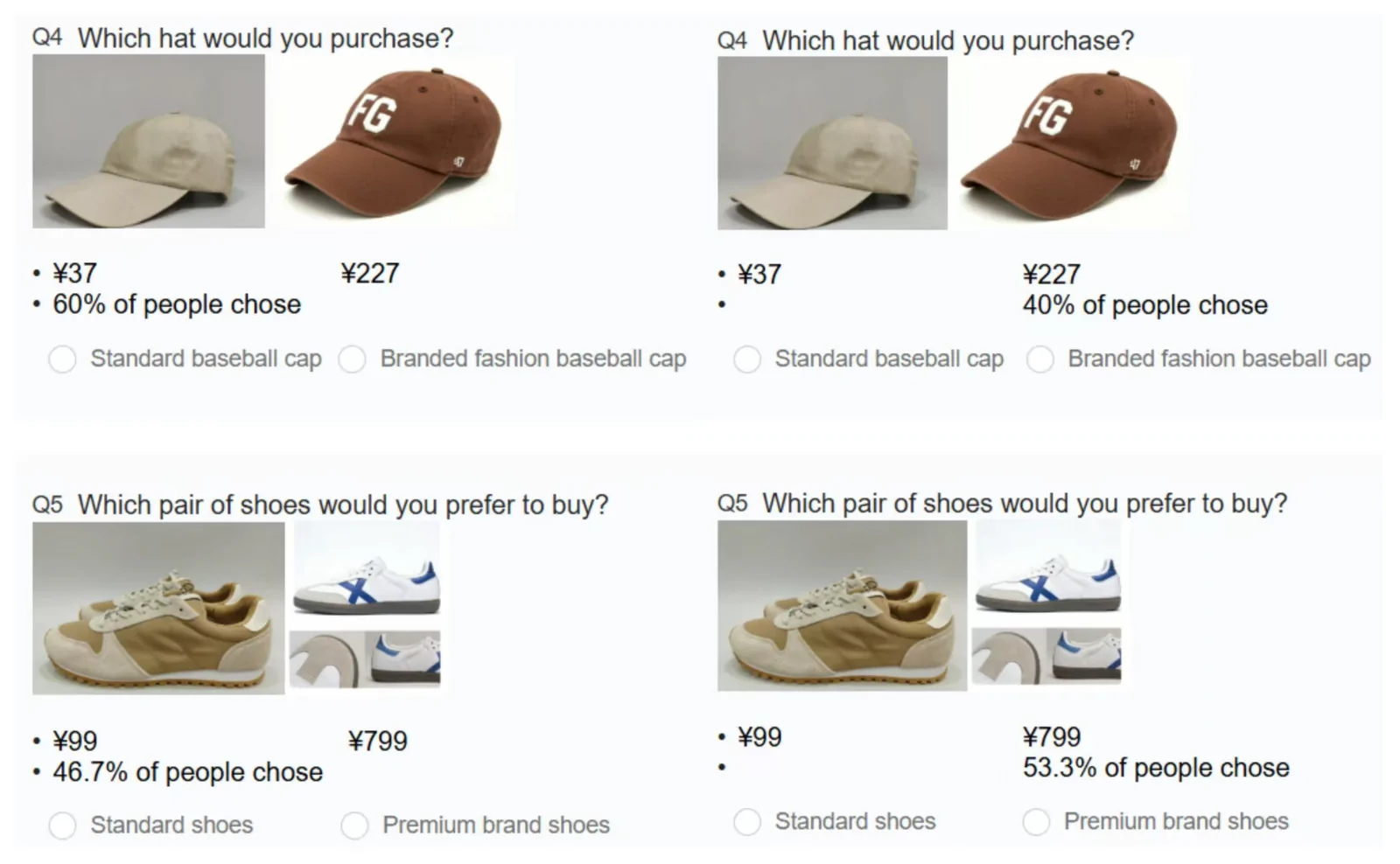
Examples of choices with information. (**left**) illustrates the cheap salience treatment; (**right**) illustrates the expensive salience treatment. Both panels present complementary information derived from the same underlying choice data.

Participants then engaged in the main consumption choice task (Questions 3–12), selecting from 10 pairs of consumption goods across various categories (fruits, beverages, shoes, hats, etc.; see Table 1). Figure 2 illustrates the visual presentation with example product pairs. Panel A (left): Cheap salience treatment showing the percentage who chose cheap products (Q3: 66.7% chose the cheaper beverage at ¥13.9; Q4: 60% chose the plain cap at ¥37). Panel B (right): Expensive salience treatment showing the percentage who chose expensive products (Q3: 33.3% chose the premium beverage at ¥33; Q4: 40% chose the Yankees cap at ¥227). These two panels illustrate complementary information of the same underlying choice data. Control group participants saw identical product images and prices but without percentage information. In all displays, the left column presented cheap products, while the right column presented expensive products (see Figure 2; *Note on Imagery: To comply with copyright regulations, the original images from the experiment have been replaced here with photos of identical angle, composition and layout taken directly by the authors*). Table 1 provides the complete list of all product pairs used in the study (see Table 1).

Before making choices, participants received instructions clarifying that (1) their anticipated income allowed them to purchase any displayed product, and (2) images and prices were for reference only; their choices represented preferences for similar products at similar price points, not necessarily the exact items shown. This disclaimer was intended to focus participants’ choices on price-tier preferences rather than on features of the specific brands and product images displayed.

Treatment group participants received additional information stating that the displayed choice percentages came from other participants facing the same income trajectory and could serve as a reference for decision-making. Participants were also informed that these percentages were obtained from a different participant pool and therefore might not fully correspond to the choices made in their own session. This clarification was intended to present the information was perceived as a reference signal rather than a deterministic forecast of others’ behavior. Depending on condition assignment, participants saw either the percentage who chose the cheap product (left column) or the percentage who chose the expensive product (right column). These percentages derived from actual pilot study data. Control group participants saw only the product pairs without any social information.

The study concluded with a post-task questionnaire covering demographics (age, gender, education), actual wealth conditions (monthly income, residence type), and perceptions about future economic growth (see Supplementary Materials).

Consumption choices were hypothetical and not financially incentivized. Participants received a fixed payment upon completion regardless of their choices. This design was adopted because the study focused on consumption decisions under hypothetical income trajectories and sought to incorporate a broad range of products and price points (from ¥13.9 to ¥7199). This choice of stimuli was intentionally designed to reflect a wide range of commodity prices and item categories in the study; however, this wide distribution precluded the practical implementation of real consumption in the experiment. Accordingly, participants made hypothetical consumption choices, reflecting a common practice in consumer behavior and psychology experiments where practical constraints often prevent the use of real consumption decisions. Prior research has shown that such hypothetical choice paradigms can provide meaningful insights into consumer preferences and decision-making under hypothetical economic scenarios (Bursztyn & Jensen, 2017; Krupka & Weber, 2013).

## 4. Main Results

Table 2 summarizes cheap product choices across all six experimental conditions and reports results for both the full 10-product sample and the nine-product sample used in the primary income-decrease analysis. Q9 was excluded from the hypothetical income-decrease condition because it was the only product for which the pilot data indicated an expensive majority; accordingly, the cheap-salience condition presented cheap-minority information for Q9 but cheap-majority information for the other nine products. The restriction therefore allows the primary comparison to focus consistently on information indicating an underlying cheap majority relative to the no-information condition and was not based on a statistical outlier-detection procedure. We refer to this nine-product sample as the analytical sample throughout the paper.^3^

As described above, treatments varied based on the provision and presentation of the pilot-derived social information, so that the experimental variation was not in the underlying information content itself, but rather in whether the information was provided and how it was presented.

### 4.1. Income Changes and Product Choice Patterns

We first examine whether income trajectory fundamentally shapes product preferences. All statistical tests reported in this section and throughout Section 4 are two-sample proportion tests. Corresponding robustness checks using independent-samples *t*-tests on participant-level means are provided in Appendix A, yielding substantively identical results.

**Figure 3:**
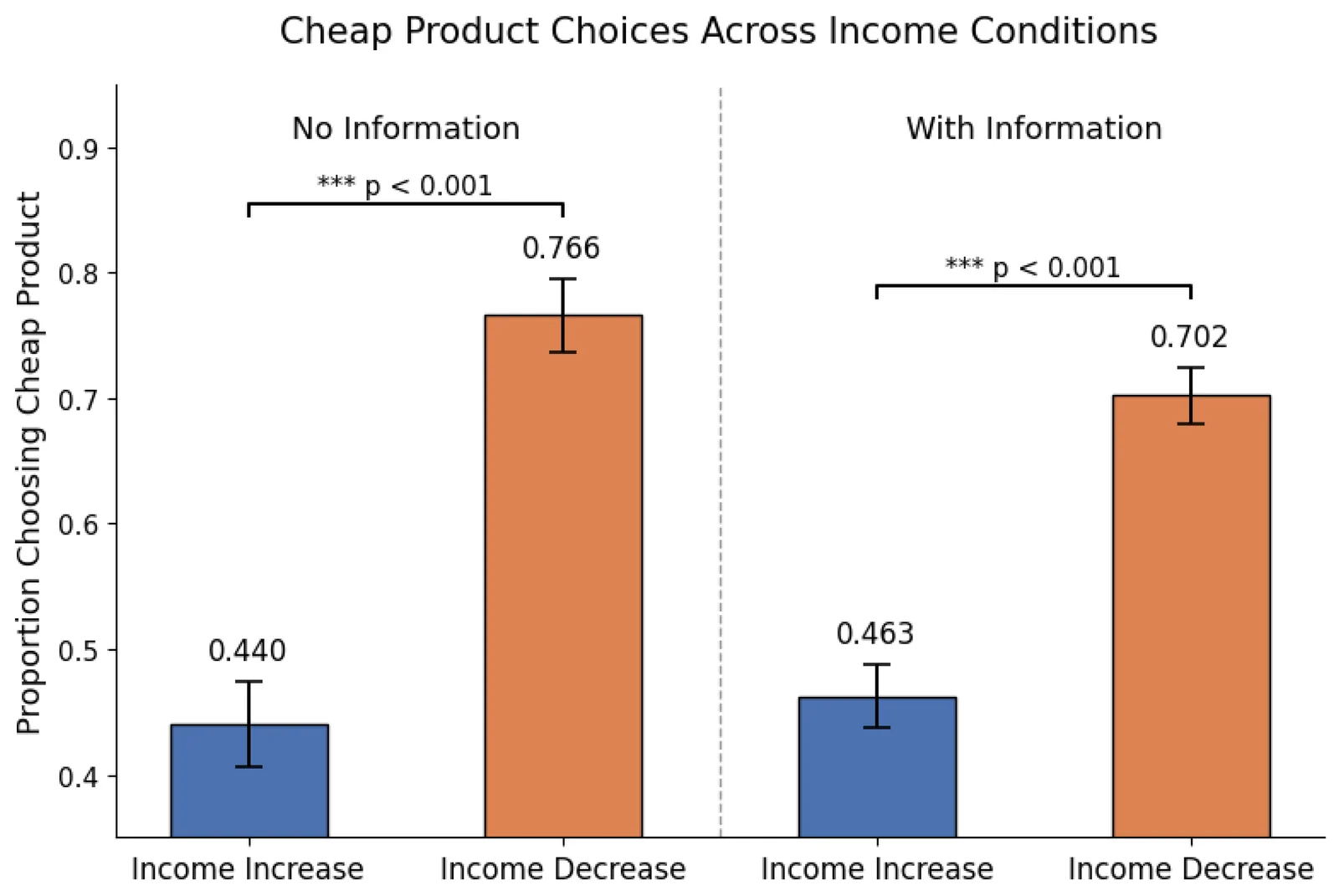
Proportion of cheap product choices by income trajectory. The left panel compares the income-increase and income-decrease groups under the no-information condition. The right panel presents the corresponding comparison for the pooled social information condition, which combines the cheap-salience and expensive-salience conditions. Error bars represent 95% confidence intervals.

Results confirm that individuals anticipating income decline choose cheap products significantly more frequently than those anticipating income increase, supporting Hypothesis 1 (See Figure 3). In the no-information condition, the proportion of cheap product choices was 0.766 among participants in the income-decrease condition, compared with 0.440 among those in the income-increase condition (p<0.001). In the pooled social information condition, the corresponding proportions were 0.702 and 0.463 for participants in the income-decrease and income-increase conditions, respectively (p<0.001).

The gap between income-declining and income-increasing groups narrows slightly in the social information conditions (0.702 − 0.463 = 0.239) relative to the no-information baseline (0.766 − 0.440 = 0.326). Notably, this aggregate pattern shows important heterogeneity. The observed difference was largely concentrated among those receiving the social information, particularly for the cheap-majority information. This indicates a complex interaction between income trajectories and the social information, which we examine in subsequent analyses (Section 4.2 and Section 4.3).

### 4.2. Social Information Effects: Evidence of Counter-Conformity

#### 4.2.1. Basic Statistical Tests

To test whether individuals under income-decline show a conformity or a counter-conformity pattern when exposed to social information (Hypothesis 2), we conducted statistical tests of proportions comparing cheap product selection rates between the no-information condition and the social information conditions within the income-decline group.

**Figure 4:**
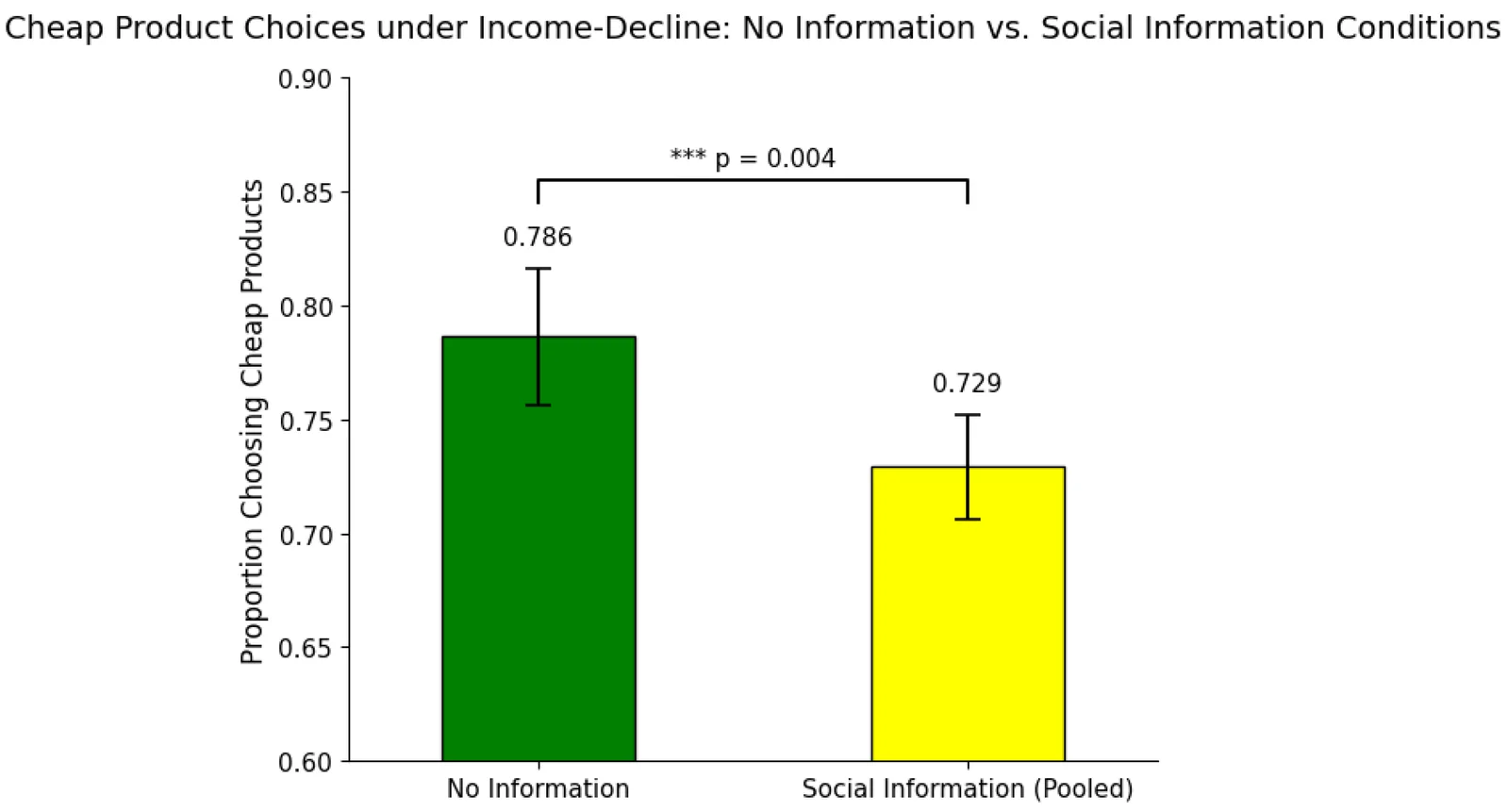
Proportion of cheap product choices under the income-decline scenario: the no-information condition versus the pooled social information condition. The pooled condition combines the cheap-salience (cheap-majority) and expensive-salience (expensive-minority) conditions. The analysis is based on the nine-product analytical sample with Q9 excluded. Error bars represent 95% confidence intervals.

Our primary analysis included all participants in the income-decrease analytical sample who were exposed to social information, pooling the cheap-salience (cheap-majority) and expensive-salience (expensive-minority) conditions. As shown in Figure 4, participants in the pooled social information conditions selected cheaper products less frequently than those receiving no information (0.729 vs. 0.786; difference =−5.7 percentage points, p=0.004, $p_{\mathrm{FDR}}=0.0082$ Cohen’s h=0.13). The difference was statistically significant but modest in standardized magnitude.^4^

**Figure 5:**
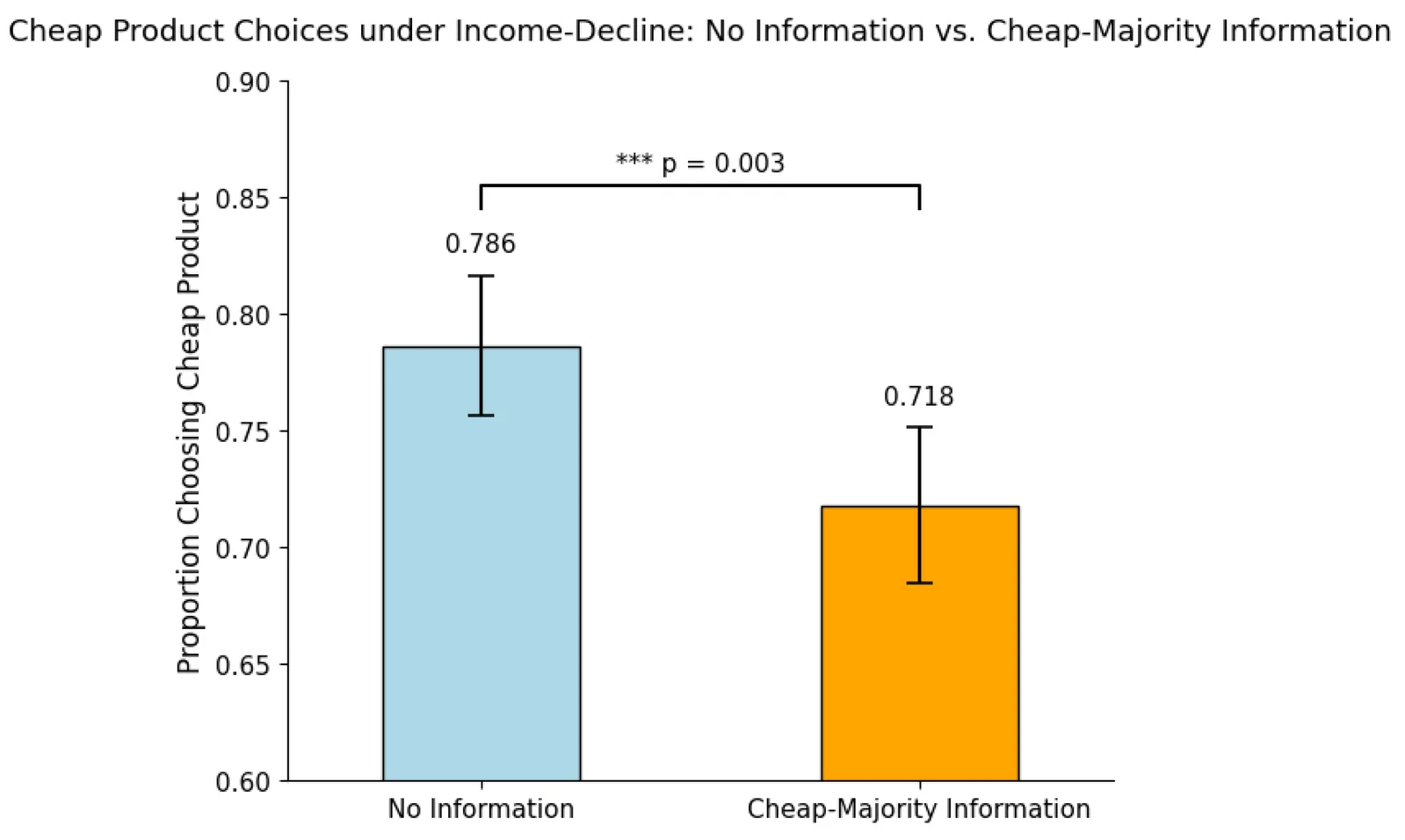
Proportion of cheap product choices under the hypothetical income-decline scenario: the no-information condition versus the cheap-salience (cheap-majority) condition. The analysis is based on the nine-product analytical sample with Q9 excluded. Error bars represent 95% confidence intervals.

We subsequently restricted the comparison to the cheap-salience (cheap-majority) and no-information conditions to examine whether the aggregate effect was associated specifically with cheap-majority information. As shown in Figure 5, the counter-conformity pattern remained statistically significant in this restricted comparison, suggesting that the aggregate result was primarily attributable to the cheap-majority information condition. The proportion for the no-information condition is identical to that reported in Figure 4 (0.786), because both analyses use the same income-decrease/no-information group as the reference.

The results show that, within the hypothetical income-decline scenario, participants exposed to social information selected the more expensive options more frequently than those receiving no information. This pattern is consistent with Hypothesis $2_{A2}$ and suggests a counter-conformity response within the experimental task.^5^

One plausible explanation is that exposure to social information triggers a pattern primarily driven by status signaling and self-concept protection. Faced with a perceived threat to their economic identity, these individuals may engage in compensatory consumption, choosing expensive products to signal financial resilience and distance themselves from the “frugal majority” (White & Dahl, 2006). By actively rejecting choices that align with their objective financial decline, they protect their self-concept from the negative connotations associated with economic constraint. This dynamic is further supported by evidence that consumer behavior is not solely driven by personal income but also by aspirational alignment with higher-status groups (Bertrand & Morse, 2016).

Furthermore, this behavior may partly stem from hedonic compensation triggered by economic anxiety. Research shows that economic anxiety heightens reliance on hedonic purchases for emotional regulation (Willis et al., 2022), and present-biased preferences are amplified under financial stress (Meier & Sprenger, 2010). Additionally, sensitivity to social comparison may spark psychological reactance: individuals may reject inexpensive options to avoid being categorized with a struggling peer group, consistent with findings that economic inequality shapes how individuals perceive their place in the social hierarchy (Kraus et al., 2017).

#### 4.2.2. Logistic Regression with Product-Pair Fixed Effects

To examine the counter-conformity pattern within a framework that accounts for differences across products, we estimated logistic regressions at the product-choice level with product-pair fixed effects. The dependent variable equals 1 if a participant selected the cheaper option within a product pair and 0 otherwise. Following our main analysis, we compared the cheap-salience group exposed to cheap-majority information with the no-information baseline within the hypothetical income-decrease condition.

The product-pair fixed effects allow the baseline propensity of selecting the cheaper option to vary across products, thereby accounting for differences in choice patterns between product categories rather than aggregating the choices into a participant-level count. Standard errors were clustered at the participant level to account for within-person correlation across the nine product choices.

As shown in Table 3, the coefficient for cheap-majority information was negative across all five specifications. It was statistically significant at the 5% level in Models 3 and 5 and at the 10% level in Models 1, 2, and 4. These results are consistent with a counter-conformity pattern after accounting for product-specific differences.

Model 5 also included participants’ perceptions of future economic growth as control variables. Relative to participants reporting a neutral economic outlook, those with a very pessimistic outlook showed a positive association with the probability of selecting the cheaper option, although the coefficient was statistically significant only at the 10% level. In contrast, optimistic and very optimistic outlooks were negatively associated with the probability of selecting the cheaper option.

#### 4.2.3. Effects of Social Information Under Economic Growth

The preceding findings suggest a counter-conformity pattern within the hypothetical income-decrease scenario: when the cheap-majority choice pattern was made salient, participants selected the more expensive alternatives more frequently. By contrast, under the hypothetical income-increase scenario, we first examined whether the pooled social information condition differed from the no-information condition. Within the four-product cheap-majority subset, the proportion of cheap product choices did not differ significantly between the no-information and pooled social information conditions (0.613 vs. 0.646, p=0.308), providing no evidence of an overall social information effect under income increase. Similarly, when the comparison was restricted to the cheap-salience (cheap-majority) condition, the proportion of cheap choices did not differ significantly from that in the no-information condition (0.607 vs. 0.613, p=0.882). These results are consistent with Hypothesis 5.

**Figure 6:**
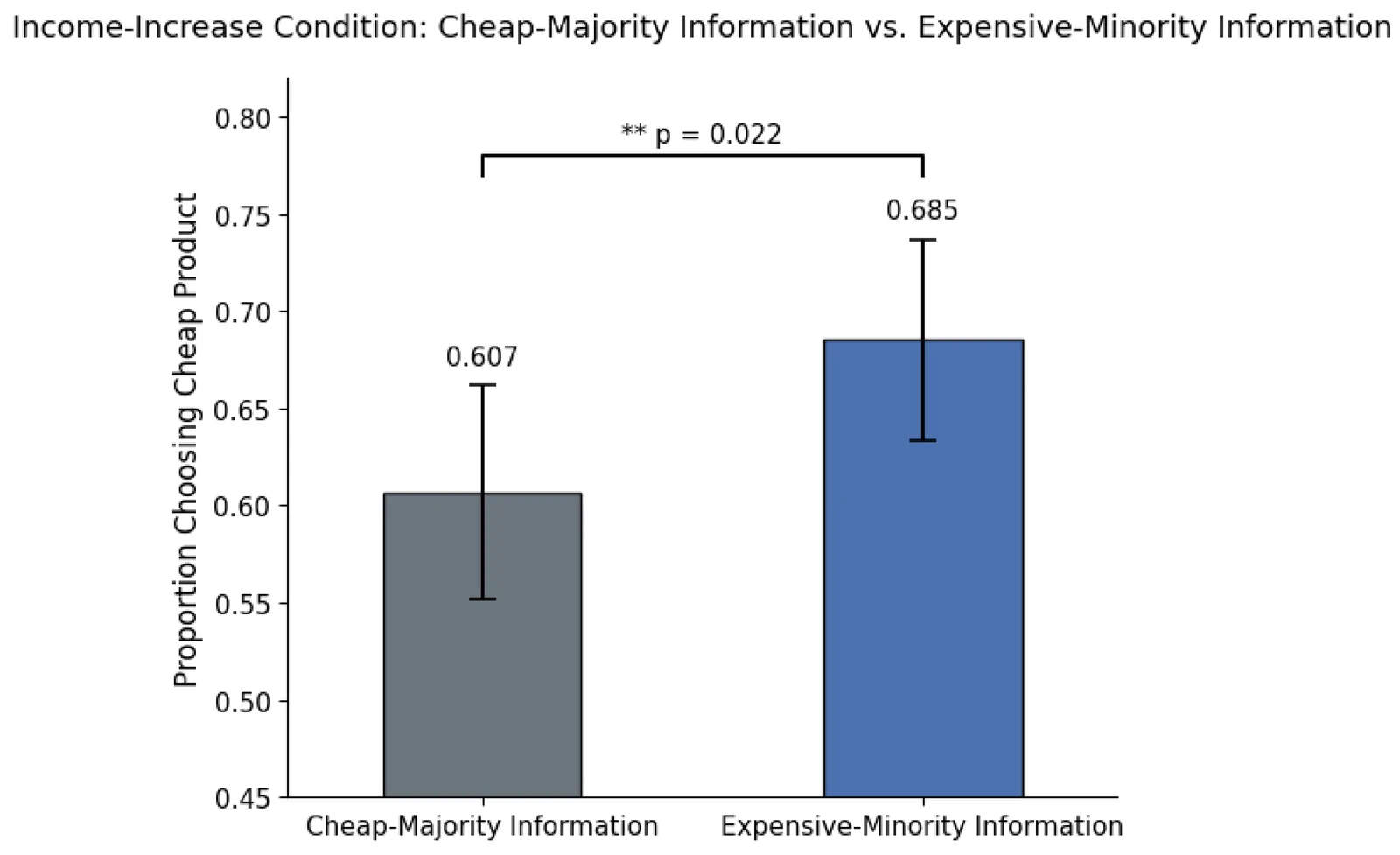
Proportion of cheap product choices under the hypothetical income-increase scenario: the cheap-salience (cheap-majority) condition versus the expensive-salience (expensive-minority) condition, restricted to Q3, Q4, Q8, and Q12. Given the directional nature of Hypothesis 6, the one-tailed *p*-value is reported (p=0.022); the corresponding two-tailed *p*-value is 0.043. Error bars represent 95% confidence intervals.

Therefore, the more relevant question in this context is whether the form of social information presentation produces differential responses within the social information conditions. Specifically, within the four-product cheap-majority subset, we compare the cheap-salience condition, which emphasizes that the majority chose the cheaper option, with the expensive-salience condition, which emphasizes that a minority chose the more expensive option. As shown in Figure 6, participants in the expensive-salience (expensive-minority) condition selected cheap products more frequently than those in the cheap-salience (cheap-majority) condition (0.685 vs. 0.607; one-tailed p=0.022, two-tailed p=0.043). This result is consistent with Hypothesis 6.

For consistency with the income-increase analysis, we also examine whether the cheap-salience (cheap-majority) and expensive-salience (expensive-minority) conditions produce differential responses under the hypothetical income-decrease scenario. The proportion of cheap product choices did not differ significantly between the cheap-salience and expensive-salience conditions (0.718 vs. 0.740; p=0.356). Thus, we found no evidence of a corresponding difference between the two forms of information presentation under income decrease.

### 4.3. Role of Information

#### 4.3.1. Baseline Conformity: Income Decline vs. Income Increase

To test whether income-declining individuals exhibit higher baseline conformity than income-rising individuals (Hypothesis 3), we estimated logistic regression models using conformity (coded 1 if participants chose the majority option, 0 otherwise) as the dependent variable. We restricted this analysis to the social information conditions to isolate the effect of income trajectories on susceptibility to social influence.

All models include demographic and socioeconomic controls to account for observable heterogeneity across participants. Residence and education controls are included but their coefficients are not reported individually as neither variable reached conventional significance thresholds; their inclusion ensures that the coefficient on the primary variable of interest (income decrease) is not confounded by these observable characteristics. The stability of the income-decrease coefficient across all five specifications confirms the robustness of our main finding. We note that the magnitude of coefficients across model specifications should not be directly compared, as the normalization of residual variance in logistic regression models renders cross-specification comparisons of coefficient size uninformative (Allison, 1999). The logistic regression suggested that income-declining participants demonstrate significantly higher conformity than income-increasing participants. This suggests that anticipated financial constraints increase reliance on social consensus as a decision heuristic, supporting Hypothesis 3 (See Table 4).

However, this finding must be interpreted cautiously due to an inherent confound in our design. Because information content was based on actual choices from our pilot study, the income-decline condition predominantly received “majority chose cheap products” information, while the distribution was more balanced in the income-increase condition. Thus, the observed higher conformity among income-declining individuals could reflect either a genuine increase in conformity propensity due to financial threat, or specific information.

We note that because social information was derived from actual pilot data, income-declining participants disproportionately received cheap-majority information, creating a mechanical correlation between income trajectory assignment and information type, a form of endogeneity discussed in (de Palma & Picard, 2005). However, our primary counter-conformity finding (Hypothesis $2_{A2}$) is identified within the income-decline condition, and is therefore unaffected by this cross-condition imbalance. The following analysis further addresses this by explicitly modeling information salience as a covariate. We address this confound by explicitly modeling information valence in Section 4.3.2.

Before proceeding to the moderation analysis, we note several control variable effects (full results in Table 4):

Participants aged 26–30 and 31–35 showed significantly higher conformity than the reference group (18–25), while those over 40 exhibited no significant difference. This pattern suggests that conformity peaks in early-to-mid age stages when social comparison may be most salient.

Higher-income participants (10,001–15,000 yuan; 20,001–25,000 yuan; >25,000 yuan) show significantly lower conformity than the lowest income bracket (<2000 yuan). After controlling for demographics, residence, education and economic perceptions, the 2001–4000 yuan bracket also showed reduced conformity, suggesting that objective financial resources reduce susceptibility to social influence.

Participants who were slightly pessimistic, slightly optimistic, or very optimistic about future economic growth showed significantly lower conformity than those with neutral expectations.

These patterns suggest both objective resources (current income) and subjective expectations (economic outlook) could reduce conformity, while mid-career age groups increases it.

#### 4.3.2. The Role of Information: Cheap Salience

To examine whether the relationship between income trajectory and observed conformity varied with the presentation of social information, we estimated logistic regression models including three primary explanatory variables: (1) anticipated income trajectory (decrease vs. increase), (2) cheap salience (coded 1 when the proportion choosing the cheaper option was displayed and 0 when the complementary proportion choosing the more expensive option was displayed), and (3) the interaction between anticipated income decrease and cheap salience. Table 5 presents nested models that progressively add control variables.

After cheap salience and its interaction with income trajectory were included, the coefficient for anticipated income decrease was not statistically significant in Models 3 and 4 and was marginally significant in Models 1, 2, and 5. Its magnitude remained relatively small across the five specifications. These results provide partial support for Hypothesis 4.

Cheap salience was negatively associated with observed conformity and reached conventional significance thresholds in Models 1–4. In the fully adjusted specification, however, the coefficient decreased in magnitude and was no longer statistically significant. This attenuation suggests sensitivity to the additional controls rather than evidence that economic outlook mediates the association between cheap salience and observed conformity.

The anticipated-income-decrease × cheap-salience interaction was not statistically significant across the five model specifications, thus providing no clear evidence that the association between cheap salience and observed conformity differed across income trajectories. The generally negative coefficient for cheap salience is consistent with a potentially broader association between cheap salience and lower observed conformity. Identity differentiation or status concerns may provide possible explanations for this pattern (Bertrand & Morse, 2016), although these mechanisms were not directly measured.

Control variables exhibited several associations with observed conformity. Participants aged 26–35 had higher estimated conformity than the 18–25 reference group in Models 4 and 5, although these coefficients were only marginally significant. Several higher-income categories were negatively associated with conformity relative to the below-2000-yuan reference category. Participants reporting slightly pessimistic or very optimistic economic outlooks also exhibited lower observed conformity than those reporting a neutral outlook.

### 4.4. Exploratory Heterogeneity Analyses Across Demographic Groups

To explore whether counter-conformity to cheap-majority information varies across demographic groups, we conducted heterogeneity analyses within the income-decline condition, comparing participants who received cheap-majority information with those who received no information. The demographic characteristics examined in these exploratory analyses—gender, income, age, and residence, were self-reported by participants as part of the survey.

#### 4.4.1. Gender Heterogeneity

The gender-stratified analysis demonstrated a statistically detectable counter-conformity pattern among female participants. Female participants exposed to cheap-majority information selected cheaper products less frequently than female participants receiving no information (69.8% vs. 77.4%; 60 vs. 58 participants, corresponding to 540 vs. 522 product-choice observations; p=0.005). Among male participants, cheap product selection was also lower in the cheap-majority information condition than in the no-information condition, although this difference was smaller and not statistically detectable (78.4% vs. 82.0%; 18 vs. 21 participants, corresponding to 162 vs. 189 product-choice observations; p=0.395). Thus, the stratified results provide evidence of a counter-conformity pattern among women in this sample. However, the gender-by-information interaction was not statistically significant, so the results do not establish that the association differed between women and men.

One possible interpretation, informed by prior research, is that responses to status-relevant consumption information may be related to identity concerns and the social signaling associated with product choices (Griskevicius et al., 2010; White & Dahl, 2006). Related economic research also indicates that gender identity norms can shape responses to status-relevant circumstances (Bertrand et al., 2015). However, the present study did not directly measure identity threat, status concerns, or the psychological mechanisms underlying the female subgroup estimate. This interpretation therefore remains tentative, and the observed gender pattern could be regarded as exploratory and hypothesis-generating for possible future work.

#### 4.4.2. Income Heterogeneity

The interaction between income group and information condition was statistically significant, suggesting contrasting patterns in two income groups, although the small number of participants within individual income brackets warrants particular caution. In the 10,001–15,000 yuan group, participants exposed to cheap-majority information selected cheaper products less frequently than those receiving no information (60.32% vs. 88.89%; 14 vs. 8 participants, corresponding to 126 vs. 72 product-choice observations; p<0.001). This within-subgroup difference is consistent with a counter-conformity pattern.

In the 15,001–20,000 yuan group, the pattern was reversed: participants exposed to cheap-majority information selected cheaper products more frequently than those receiving no information (80.2% vs. 65.3%; 9 vs. 8 participants, corresponding to 81 vs. 72 product-choice observations; p=0.037). The contrasting estimates suggest that income could relevant to how participants respond to cheap-majority information. However, given the small subgroup sizes, and exploratory nature of the analysis, these results do not establish income as a boundary condition or identify the psychological mechanisms underlying the differences.

#### 4.4.3. Age Heterogeneity

Exploratory age-stratified analyses suggested different patterns across age groups. Among participants aged 35 and below, exposure to cheap-majority information was associated with less frequent selection of cheaper products (60 vs. 55 participants, corresponding to 540 vs. 495 product-choice observations; z=2.50, p=0.012), consistent with a counter-conformity pattern. Among participants aged 36–40, the estimated difference was in the opposite direction, although it was not statistically detectable (10 vs. 15 participants, corresponding to 90 vs. 135 product-choice observations).

The interaction between age group and information condition was not statistically significant. Accordingly, the contrasting subgroup estimates do not establish that responses to cheap-majority information differed by age. The pattern may nevertheless be considered in light of research showing that product choices can serve identity-signaling and differentiation functions (Berger & Heath, 2007), as well as evidence that decision-making tendencies may vary across age groups and decision domains (Weber et al., 2002). However, the present study did not directly measure age-related differences in information processing. These interpretations therefore remain tentative.

#### 4.4.4. Residential-Area Heterogeneity

Exploratory analyses by residential area type showed a statistically detectable counter-conformity pattern among town residents. Participants in towns who received cheap-majority information selected cheaper products less frequently than those receiving no information (11 vs. 14 participants, corresponding to 99 vs. 126 product-choice observations; z=3.38, p=0.001). Among city residents, cheap product selection was also lower in the cheap-majority information condition than in the no-information condition (72.7% vs. 76.5%; 66 vs. 61 participants, corresponding to 594 vs. 549 product-choice observations), although the difference was not statistically detectable in a two-tailed test (p=0.143).

The residence-by-information interaction was not statistically significant. The subgroup estimates therefore provide evidence of a counter-conformity pattern among town residents in this sample but do not establish that the association differed between town and city residents. Interpretation is further limited by the small number of town residents.

To examine whether income composition might contribute to the descriptive residence pattern, we estimated models including the interaction between residence and information condition before and after controlling for participant income (N=157). The town-specific interaction was not statistically significant in either specification (without income controls: β=−0.734, p=0.503; with income controls: β=−0.505, p=0.682) and attenuated toward zero after income was included. Town residents also had a lower mean income-group value than city residents (3.40 vs. 4.69), although the difference did not reach conventional statistical significance, $\chi^{2}\left(8\right)=14.51$, p=0.069. These results are consistent with the possibility that income composition contributes to the descriptive residence pattern, but they do not establish that income accounts for that pattern or that residence independently moderates the association.

**Figure 7:**
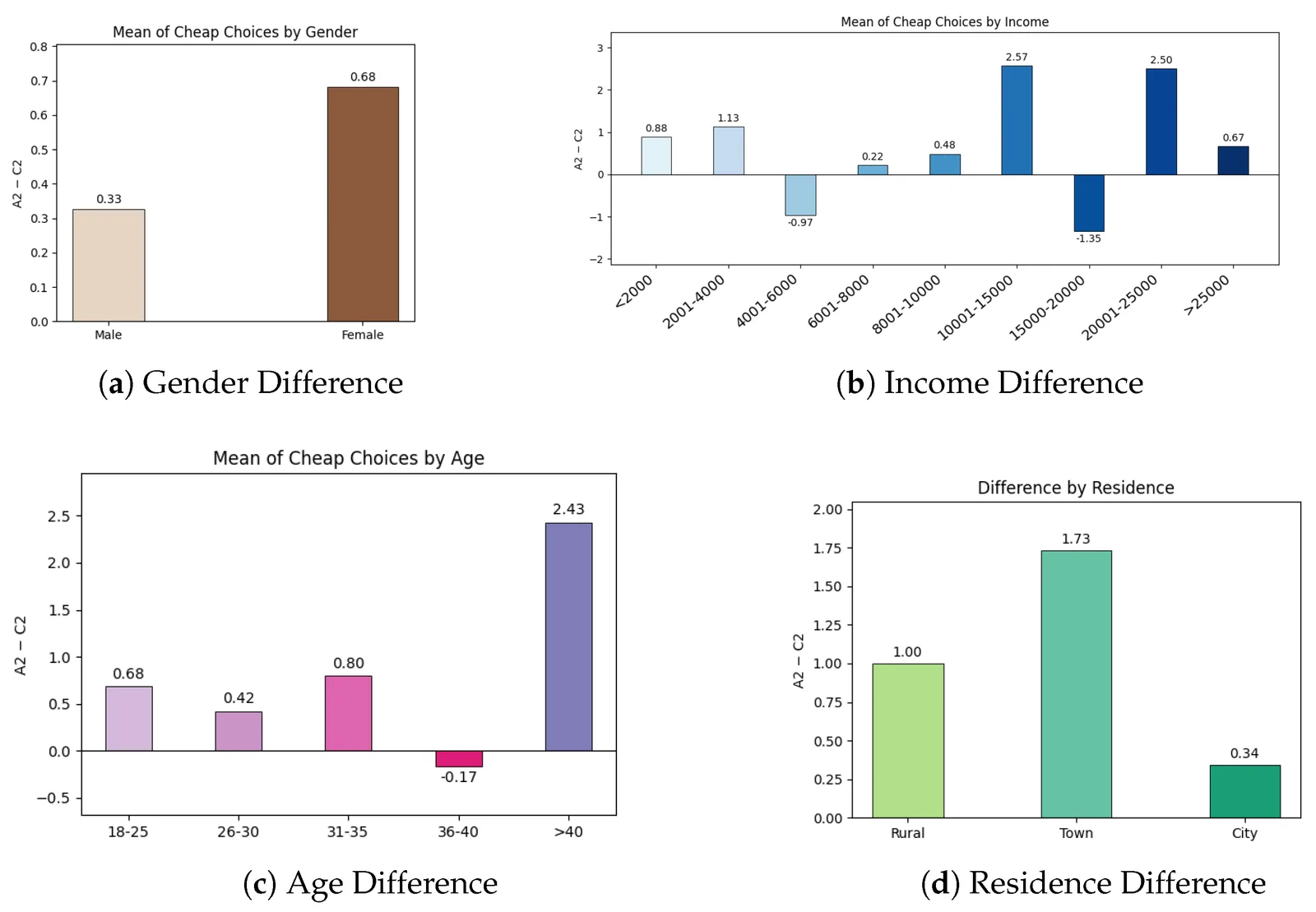
Heterogeneous effects: (**a**) gender difference; (**b**) income difference; (**c**) age difference; (**d**) residence difference.

The distribution of these heterogeneous effects across subgroups is summarized in Figure 7. The Y-axis represents the difference in the mean proportion of cheap product choices between the no-information and cheap-salience (cheap-majority) conditions under the hypothetical income-decrease scenario (mean for no information minus mean for cheap-majority information). Larger positive values indicate a more pronounced counter-conformity pattern, whereas negative values indicate a shift toward conformity with the cheap-majority information.

## 5. Summary and Discussion

This paper examines how income trajectories interact with social information to shape consumption choices under economic uncertainty. Using a randomized online experiment, we manipulate expected income trajectories (increase versus decrease) and provide participants with social information that the majority choice was either cheap or expensive. This design allows us to causally identify how social signals affect consumption decisions conditional on anticipated economic conditions.

Under anticipated income decline, participants exposed to social information indicating an underlying cheap majority selected expensive products more frequently than those receiving no information. This result is consistent with a counter-conformity pattern rather than increased frugal choice. Under anticipated income increase, no statistically significant overall difference was observed between the social information and no-information conditions. Within the social information conditions, however, participants receiving expensive-minority information selected cheap products more frequently than those receiving cheap-majority information. Separately, we examined conformity, defined as choosing the majority option, among participants receiving social information. Cheap salience was negatively associated with observed conformity in most model specifications; however, its interaction with anticipated income decrease was not statistically significant. The results therefore do not establish that this association under different anticipated income trajectories.

Our findings contribute to the literature on consumption under economic insecurity and social influence by showing that pessimistic income trajectories alter not only price sensitivity but also the interpretation of social information. While existing work emphasizes frugal conformity during downturns, our results document a systematic reversal when cheap consumption is socially emphasized, consistent with identity- and status-related mechanisms. More broadly, the paper adds to the reference-dependence literature by highlighting that the social meaning attached to prices can dominate their instrumental role in shaping choices.

These findings carry important implications for consumer financial well-being and policy design. When individuals under economic stress are exposed to information about others’ consumption choices, the resulting counter-conformity response may lead them to overspend relative to their means, a pattern that could deepen financial strain rather than alleviate it. Accordingly, information-based interventions that promote frugality by emphasizing cheap-majority behavior may be ineffective or even counterproductive during economic downturns. Policy communication and consumer guidance should therefore account for the social and expressive meanings conveyed by price-related messages, particularly for economically vulnerable populations.

Several limitations of the current design merit acknowledgment, each of which also points to productive directions for future research. First, the consumption choices were hypothetical and non-incentivized. This design allowed anticipated income trajectories and social information to be manipulated consistently and has precedent in related research (Bursztyn & Jensen, 2017). The absence of real financial consequences limits generalizability to consequential purchasing behavior. Real stakes could strengthen the observed pattern by heightening financial anxiety or weaken it if budget constraints outweigh status concerns. The findings therefore concern stated preferences under anticipated income changes, and future incentive-compatible or field studies could examine whether the pattern extends to actual purchases. Second, our design exogenously provides social information to participants, enabling clean identification of its effect on consumption choices, an advantage over observational settings where social information is endogenous to consumer preferences. A natural extension would be field studies examining naturally occurring social information exposure to further establish external validity. Third, participants imagined future income trajectories rather than responding to realized income changes. The effectiveness of this manipulation is supported by the manipulation check (income-decreasing participants reported significantly lower satisfaction: M=2.56 vs. M=7.76, p<0.001), consistent with established practice in experimental studies of economic anticipations (Beck et al., 2016). Future research using naturally occurring income variation or field experiments would provide complementary evidence. Fourth, the cheap product was consistently presented in the left column across all displays. Importantly, position bias alone cannot account for our main finding, as the counter-conformity effect is specific to the income-decline condition and absent under the income-increase condition. Future research could randomize product presentation order as standard practice. Fifth, all participants were recruited via the Chinese online platform Credamo. Status-conformity dynamics and the social meanings attached to consumption choices may vary across cultural contexts, and the generalizability of our findings beyond the Chinese setting remains to be established. Future cross-cultural research would help clarify the boundary conditions of these effects.

## Figures and Tables

**Table 1:** Product pairs and pilot selection percentages.

Q#	Category	Cheap (Price)	Expensive (Price)	Income Decrease		Income Increase	
				Cheap%	Expensive%	Cheap%	Expensive %
Q3	Beverage	Domestic chain (¥13.9)	International chain (¥33)	100.0	0.0	66.7	33.3
Q4	Hat	Basic (¥37)	Branded (¥227)	86.7	13.3	60.0	40.0
Q5	Shoes	Standard (¥99)	Premium (¥799)	76.7	23.3	46.7	53.3
Q6	Meal	Box meal (¥15)	Stir-fry (¥27)	56.7	43.3	26.7	73.3
Q7	Fruit	Loose (¥15)	Premium (¥45)	83.3	16.7	43.3	56.7
Q8	Sports	Basic racket (¥114)	Carbon (¥560)	86.7	13.3	66.7	33.3
Q9	Cup	Practical (¥20)	Premium (¥84)	46.7	53.3	16.7	83.3
Q10	Shampoo	Basic (¥39)	Functional (¥120)	63.3	36.7	23.3	76.7
Q11	Skincare	Basic (¥122)	Premium (¥530)	70.0	30.0	33.3	66.7
Q12	Phone	Domestic (¥4775)	International (¥7199)	86.7	13.3	70.0	30.0

**Table 2:** Cheap product choices by income trajectory and information condition.

Information Condition	Income Trajectory	Total Cheap Choices	Total Responses	Proportion
Panel A. Full sample: all 10 products				
No Information	Increase	352	800	0.440
No Information	Decrease	605	790	0.766
Cheap Salience	Increase	340	750	0.453
Cheap Salience	Decrease	537	780	0.688
Expensive Salience	Increase	363	770	0.471
Expensive Salience	Decrease	565	790	0.715
Panel B. Income Decrease cheap-majority product (analytical sample): Q9 excluded				
No Information	Decrease	559	711	0.786
Cheap Salience (Cheap-Majority)	Decrease	504	702	0.718
Expensive Salience (Expensive-Minority)	Decrease	526	711	0.740
Social Information (Pooled)	Decrease	1030	1413	0.729
Panel C. Income Increase cheap-majority product: Q3, Q4, Q8, and Q12				
No Information	Increase	196	320	0.613
Cheap Salience (Cheap-Majority)	Increase	182	300	0.607
Expensive Salience (Expensive-Minority)	Increase	211	308	0.685
Social Information (Pooled)	Increase	393	608	0.646

Note: The final valid sample consists of 468 participants: 80 in the income-increase/no-information condition, 79 in the income-decrease/no-information condition, 75 in the income-increase/cheap-salience condition, 78 in the income-decrease/cheap-salience condition, 77 in the income-increase/expensive-salience condition, and 79 in the income-decrease/expensive-salience condition. The cheap-salience and expensive-salience conditions displayed the proportions choosing the cheap and expensive options, respectively; these proportions were complementary presentations of the same underlying pilot choice data. Total responses refer to valid product-choice observations rather than the number of participants. Panel A reports choices across all 10 products. Panel B reports the nine-product analytical sample under the income-decrease trajectory with Q9 excluded. Because Q9 was the only product for which the pilot data indicated an expensive majority under income decline, the remaining nine products presented cheap-majority information in the cheap-salience condition and the complementary expensive-minority information in the expensive-salience condition. Panel C restricts the income-increase sample to Q3, Q4, Q8, and Q12, the four products for which the pilot data indicated a cheap majority; accordingly, these products likewise presented cheap-majority information in the cheap-salience condition and complementary expensive-minority information in the expensive-salience condition. In Panels B and C, “Social Information (Pooled)” combines the corresponding cheap-majority and expensive-minority information conditions. Proportions are calculated by dividing the total number of cheap product choices by the total number of valid product-choice observations within each panel.

**Table 3:** Logistic regression with product-pair fixed effects: Cheap-majority information and cheap product choices under anticipated income decline.

VARIABLES	(1)	(2)	(3)	(4)	(5)
	Cheap Choice	Cheap Choice	Cheap Choice	Cheap Choice	Cheap Choice
Cheap-Majority Information	−0.401 *	−0.390 *	−0.464 **	−0.411 *	−0.458 **
	(0.218)	(0.218)	(0.213)	(0.213)	(0.219)
Economic Outlook:					
Very Pessimistic					1.929 *
					(1.039)
Pessimistic					−0.042
					(0.396)
Slightly Pessimistic					−0.296
					(0.317)
Slightly Optimistic					−0.266
					(0.350)
Optimistic					−1.226 **
					(0.620)
Very Optimistic					−1.713 ***
					(0.452)
Fixed Effects and Controls Included:					
Product-pair fixed effects	Yes	Yes	Yes	Yes	Yes
Gender	No	Yes	Yes	Yes	Yes
Age	No	No	Yes	Yes	Yes
Income	No	No	No	Yes	Yes
Residential area	No	No	No	Yes	Yes
Education	No	No	No	No	Yes
Constant	0.393 **	0.692 ***	0.587 **	1.136 *	2.011 **
	(0.194)	(0.257)	(0.297)	(0.639)	(0.932)
Product-choice observations	1413	1413	1413	1413	1413
Participants	157	157	157	157	157
Log-likelihood	−724.683	−721.296	−713.863	−700.819	−675.508
AIC	1469.366	1464.591	1457.727	1451.639	1419.015
McFadden $R^{2}$	0.084	0.088	0.098	0.114	0.146

Standard errors clustered at the participant level in parentheses; *** p<0.01, ** p<0.05, * p<0.10. Note:  The dependent variable equals 1 for selection of the cheaper option and 0 for selection of the more expensive option within each product pair. The sample includes 79 participants in the no-information condition and 78 participants in the cheap-salience (cheap-majority) condition under the hypothetical income-decrease scenario, yielding 1413 product-choice observations from 157 participants. Q9 is excluded because the primary analysis compares cheap-majority information with no information, whereas Q9 was the only product for which the corresponding pilot data indicated an expensive majority under the hypothetical income-decrease scenario. All models include product-pair fixed effects. Reported estimates are logit coefficients rather than odds ratios. Average marginal effects for cheap-majority information relative to no information were −0.068 (SE=0.037, p=0.065) in Model 1, −0.066 (SE=0.037, p=0.073) in Model 2, −0.078 (SE=0.036, p=0.029) in Model 3, −0.067 (SE=0.035, p=0.052) in Model 4, and −0.072 (SE=0.034, p=0.036) in Model 5. “Yes” indicates that the corresponding fixed effects or controls are included, with their coefficients omitted for brevity; “No” indicates that they are not included. The reference categories are “No Information” for the information condition, Q6 (Meal) for the product-pair fixed effects, and “Neutral” for economic outlook. All reported *p*-values are two-tailed and unadjusted.

**Table 4:** Logistic regression—baseline conformity by income trajectories with info.

VARIABLES	(1)	(2)	(3)	(4)	(5)
	Conformity	Conformity	Conformity	Conformity	Conformity
Income Decrease	0.257 ***	0.265 ***	0.249 ***	0.231 ***	0.274 ***
	(0.087)	(0.087)	(0.088)	(0.089)	(0.088)
Gender: Female		−0.117	−0.116	−0.091	−0.068
		(0.103)	(0.106)	(0.103)	(0.103)
Age: 26–30			0.128	0.233 *	0.243 *
			(0.122)	(0.127)	(0.125)
Age: 31–35			0.099	0.249 **	0.256 **
			(0.108)	(0.125)	(0.128)
Age: 36–40			0.044	0.135	0.106
			(0.166)	(0.175)	(0.181)
Age: >40			−0.140	−0.004	0.028
			(0.157)	(0.159)	(0.174)
Income: 2001–4000				−0.302 *	−0.345 **
				(0.162)	(0.161)
Income: 4001–6000				−0.144	−0.158
				(0.177)	(0.176)
Income: 6001–8000				−0.069	−0.058
				(0.163)	(0.166)
Income: 8001–10,000				−0.121	−0.126
				(0.181)	(0.184)
Income: 10,001–15,000				−0.562 ***	−0.565 ***
				(0.174)	(0.175)
Income: 15,001–20,000				−0.235	−0.306
				(0.236)	(0.240)
Income: 20,001–25,000				−0.594 **	−0.646 **
				(0.255)	(0.255)
Income: >25,000				−0.773 **	−0.705 **
				(0.311)	(0.283)
Economic Outlook: Very Pessimistic					−0.423
					(0.298)
Economic Outlook: Pessimistic					−0.229
					(0.160)
Economic Outlook: Slightly Pessimistic					−0.226 *
					(0.125)
Economic Outlook: Slightly Optimistic					−0.232 *
					(0.141)
Economic Outlook: Optimistic					−0.068
					(0.159)
Economic Outlook: Very Optimistic					−0.862 ***
					(0.226)
Constant	0.639 ***	0.721 ***	0.686 ***	0.814 ***	0.950 ***
	(0.050)	(0.093)	(0.116)	(0.221)	(0.327)
Controls Included:					
Residence	No	No	No	Yes	Yes
Education	No	No	No	No	Yes
Observations	3090	3090	3090	3090	3090
Log-likelihood	−1924.82	−1923.94	−1921.80	−1905.49	−1897.94
AIC	3853.65	3853.89	3857.60	3844.98	3847.89
McFadden $R^{2}$	0.003	0.003	0.004	0.013	0.017

Standard errors clustered at participant level in parentheses; *** *p* < 0.01, ** *p* < 0.05, * *p* < 0.1. Note: The sample includes all participants in the social information conditions across both income trajectories. The final sample consists of 309 unique participants: 75 in the income-increase/cheap-salience condition, 77 in the income-increase/expensive-salience condition, 78 in the income-decrease/cheap-salience condition, and 79 in the income-decrease/expensive-salience condition. Each participant made 10 product choices, yielding 3090 question-level observations. The income-increase group includes 152 participants from the two social information conditions (152×10=1520 observations), while the income-decrease group includes 157 participants from the corresponding social information conditions (157×10=1570 observations). Residence and education are included as controls in Models 4 and 5, respectively, but their coefficients are not reported because neither reached conventional levels of statistical significance. The reference categories are “Income Increase,” “Age 18–25,” “Income below 2000 yuan,” “Neutral economic outlook,” and “Vocational school”.

**Table 5:** Logistic regression–role of cheap salience in conformity.

VARIABLES	(1)	(2)	(3)	(4)	(5)
	Conformity	Conformity	Conformity	Conformity	Conformity
Income Decrease	0.189 *	0.199 *	0.176	0.164	0.209 *
	(0.111)	(0.112)	(0.115)	(0.116)	(0.117)
Cheap Salience	−0.197 **	−0.190 *	−0.190 *	−0.181 *	−0.153
	(0.098)	(0.098)	(0.098)	(0.100)	(0.101)
Income Decrease × Cheap Salience	0.136	0.130	0.144	0.131	0.123
	(0.173)	(0.173)	(0.174)	(0.171)	(0.173)
Gender: Female		−0.108	−0.105	−0.084	−0.061
		(0.102)	(0.106)	(0.103)	(0.102)
Age: 26–30			0.131	0.229 *	0.240 *
			(0.122)	(0.127)	(0.126)
Age: 31–35			0.090	0.228 *	0.238 *
			(0.109)	(0.126)	(0.129)
Age: 36–40			0.029	0.107	0.079
			(0.166)	(0.175)	(0.181)
Age: >40			−0.139	−0.016	0.015
			(0.154)	(0.155)	(0.171)
Income: 2001–4000				−0.311 *	−0.352 **
				(0.162)	(0.161)
Income: 4001–6000				−0.139	−0.156
				(0.178)	(0.177)
Income: 6001–8000				−0.068	−0.063
				(0.167)	(0.173)
Income: 8001–10,000				−0.124	−0.126
				(0.182)	(0.186)
Income: 10,001–15,000				−0.550 ***	−0.555 ***
				(0.174)	(0.176)
Income: 15,001–20,000				−0.198	−0.276
				(0.238)	(0.243)
Income: 20,001–25,000				−0.578 **	−0.632 **
				(0.251)	(0.256)
Income: >25,000				−0.748 **	−0.693 **
				(0.317)	(0.289)
Economic Outlook: Very Pessimistic					−0.418
					(0.302)
Economic Outlook: Pessimistic					−0.221
					(0.161)
Economic Outlook: Slightly Pessimistic					−0.228 *
					(0.124)
Economic Outlook: Slightly Optimistic					−0.224
					(0.143)
Economic Outlook: Optimistic					−0.061
					(0.156)
Economic Outlook: Very Optimistic					−0.838 ***
					(0.225)
Constant	0.738 ***	0.810 ***	0.777 ***	0.892 ***	1.018 ***
	(0.068)	(0.100)	(0.123)	(0.233)	(0.333)
Controls Included:					
Residence	No	No	No	Yes	Yes
Education	No	No	No	No	Yes
Observations	3090	3090	3090	3090	3090
Log-likelihood	−1923.00	−1922.26	−1920.19	−1904.07	−1896.99
AIC	3854.00	3854.51	3858.39	3846.14	3849.97
McFadden $R^{2}$	0.004	0.004	0.005	0.014	0.017

Standard errors clustered at the participant level are reported in parentheses. *** p<0.01, ** p<0.05, * p<0.10. Note: The sample includes participants in the cheap-salience and expensive-salience conditions under both income trajectories, yielding 3090 question-level observations from 309 unique participants (309×10 product choices). Residence and education are included as controls in Models 4 and 5, respectively, but their coefficients are not reported because neither reached conventional levels of statistical significance. The reference categories are “Income Increase,” “Expensive Salience,” “Age 18–25,” “Income below 2000 yuan,” “Neutral Economic Outlook,” and “Vocational School”.

**Figure A1:**
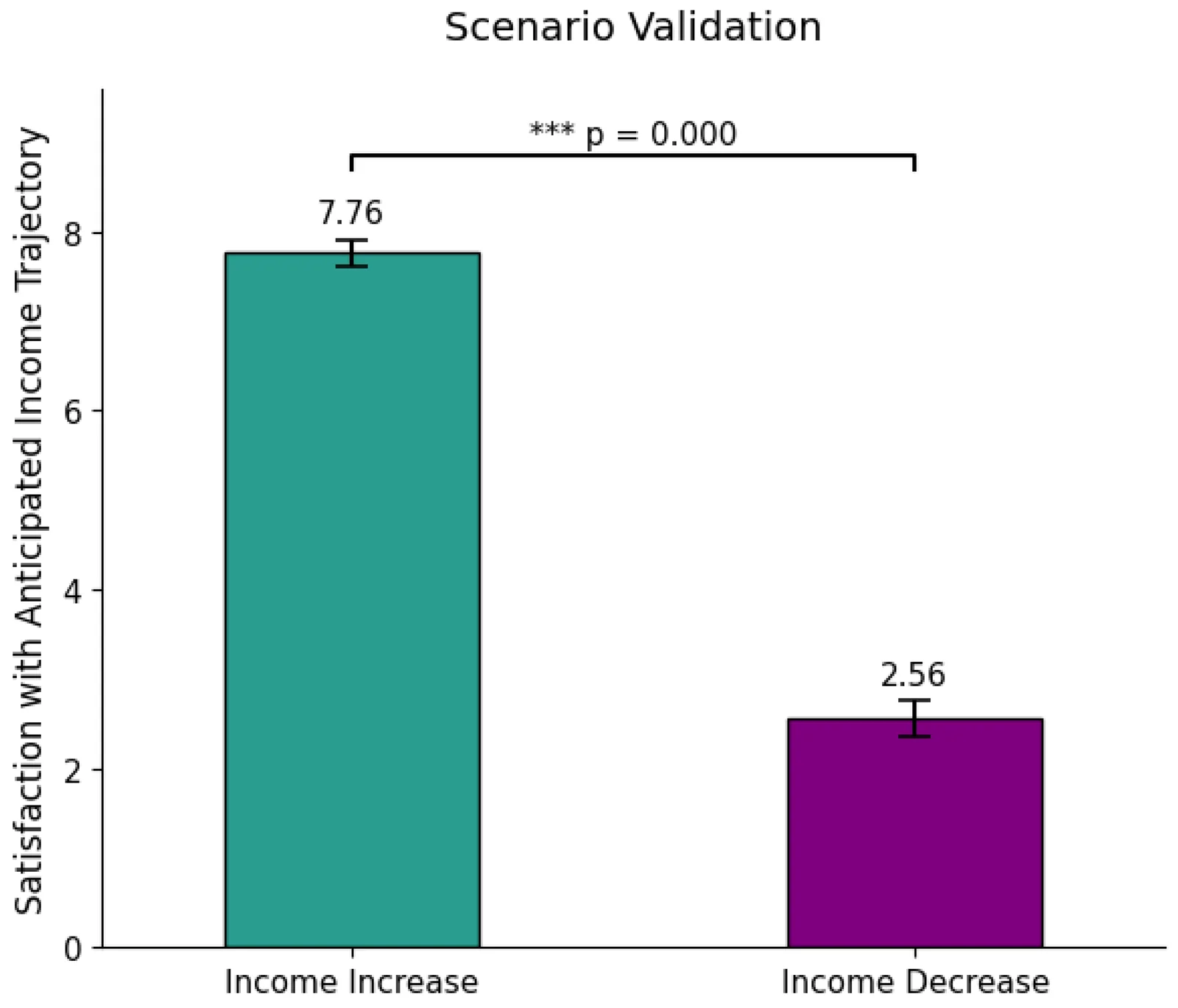
Scenario validation based on Q1 satisfaction ratings by income-trajectory condition. Across all information conditions, participants assigned to the hypothetical income-decrease scenario reported lower satisfaction than those assigned to the hypothetical income-increase scenario (M=2.56 vs. M=7.76, t(466)=39.97, p<0.001). This difference indicates that participants’ self-reported evaluations varied in the intended direction across the two income-trajectory scenarios.

**Figure A2:**
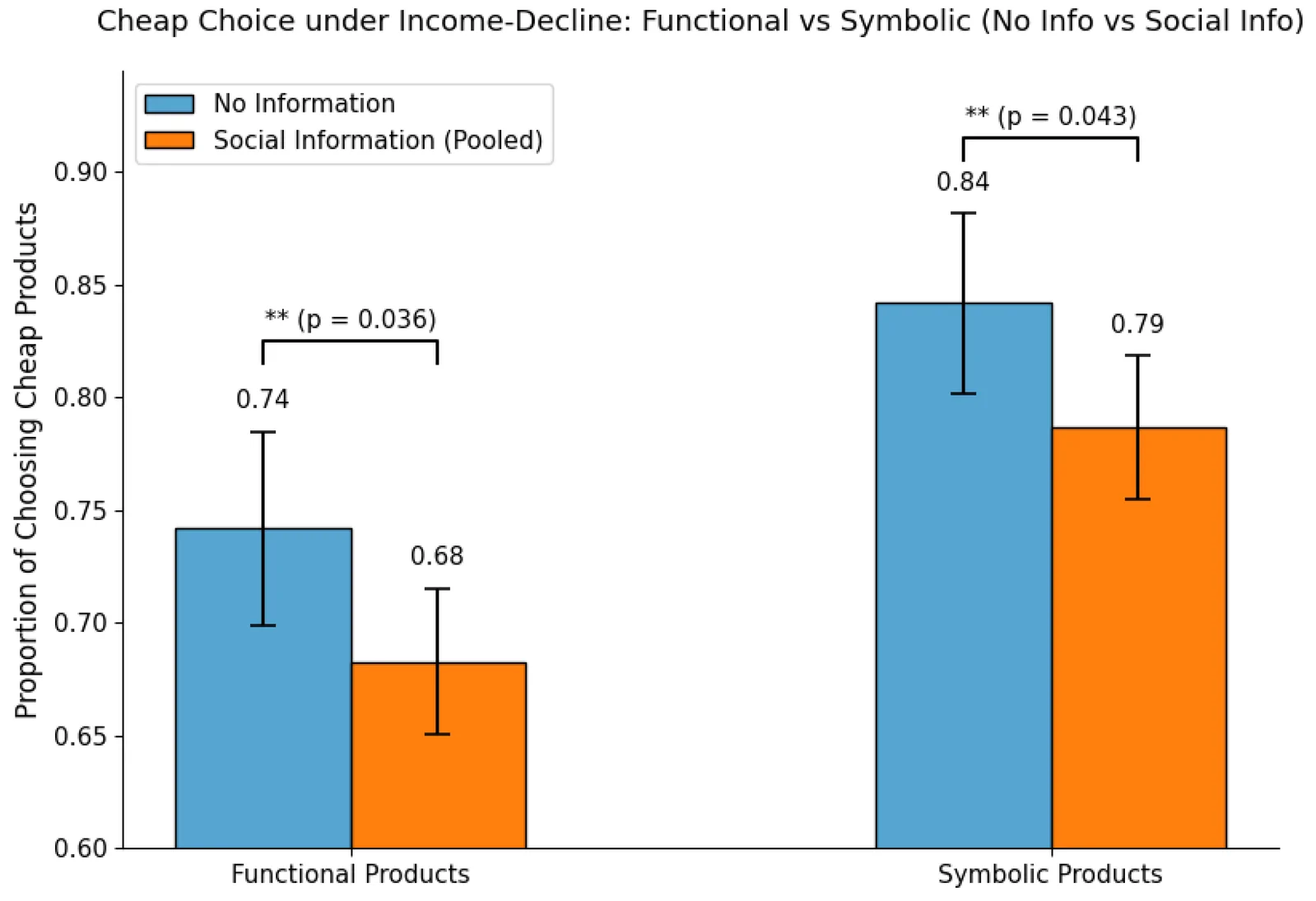
Proportion of cheap product choices under the hypothetical income-decrease scenario, shown separately for functional and symbolic products. Functional products comprise Q3, Q6, Q7, Q10, and Q11, whereas symbolic products comprise Q4, Q5, Q8, and Q12. Q9 is excluded from this analysis. “Social Information (Pooled)” combines the cheap-salience and expensive-salience conditions. Error bars represent 95% confidence intervals.

**Figure A3:**
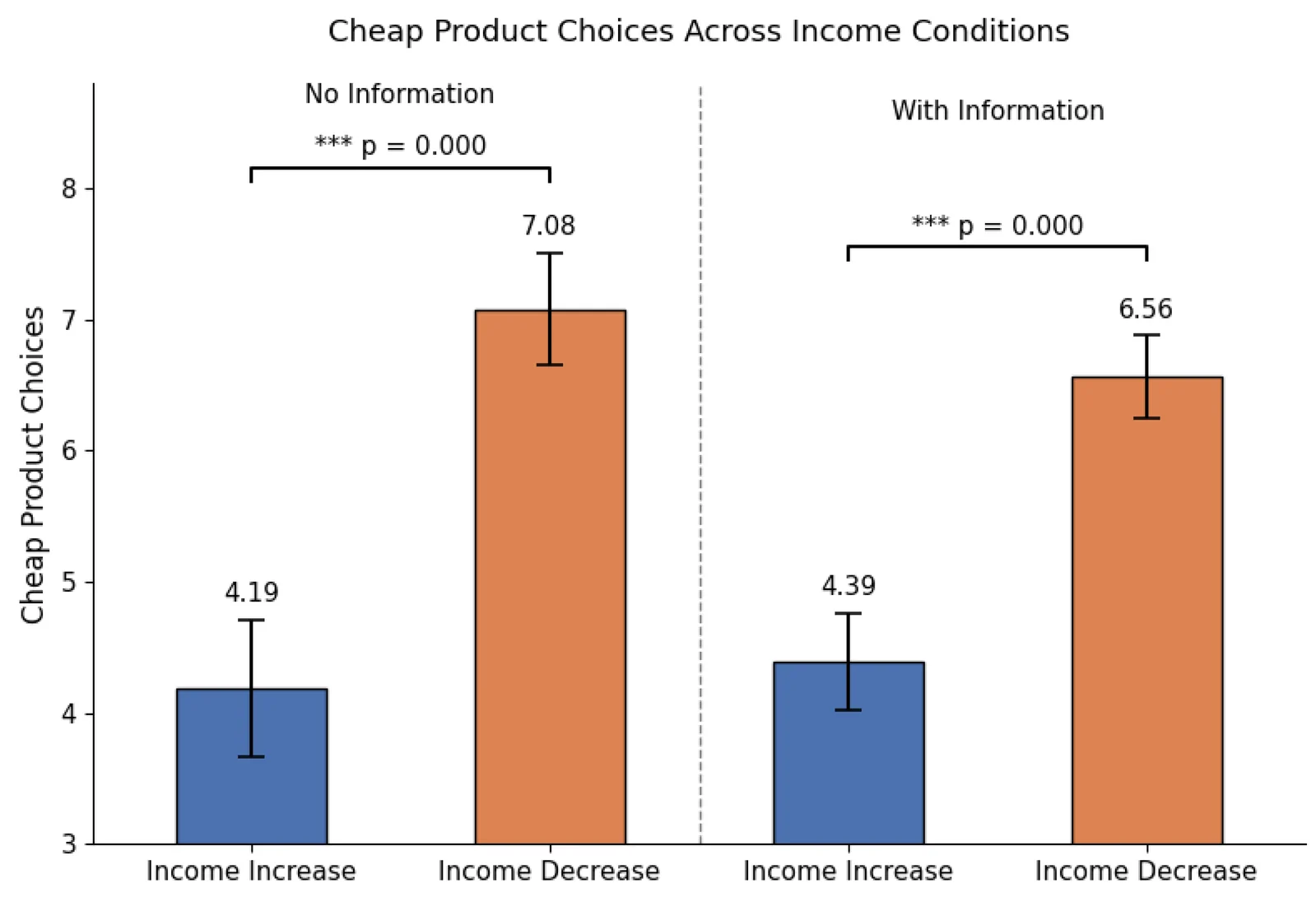
Mean number of cheap product choices by income trajectory. The no-information comparison includes participants assigned to the income-increase and income-decrease scenarios without social information. The social information comparison includes the corresponding income-trajectory groups after pooling the cheap-salience and expensive-salience conditions. Results are based on independent-samples *t*-tests using participant-level choice totals. Error bars represent 95% confidence intervals.

**Figure A4:**
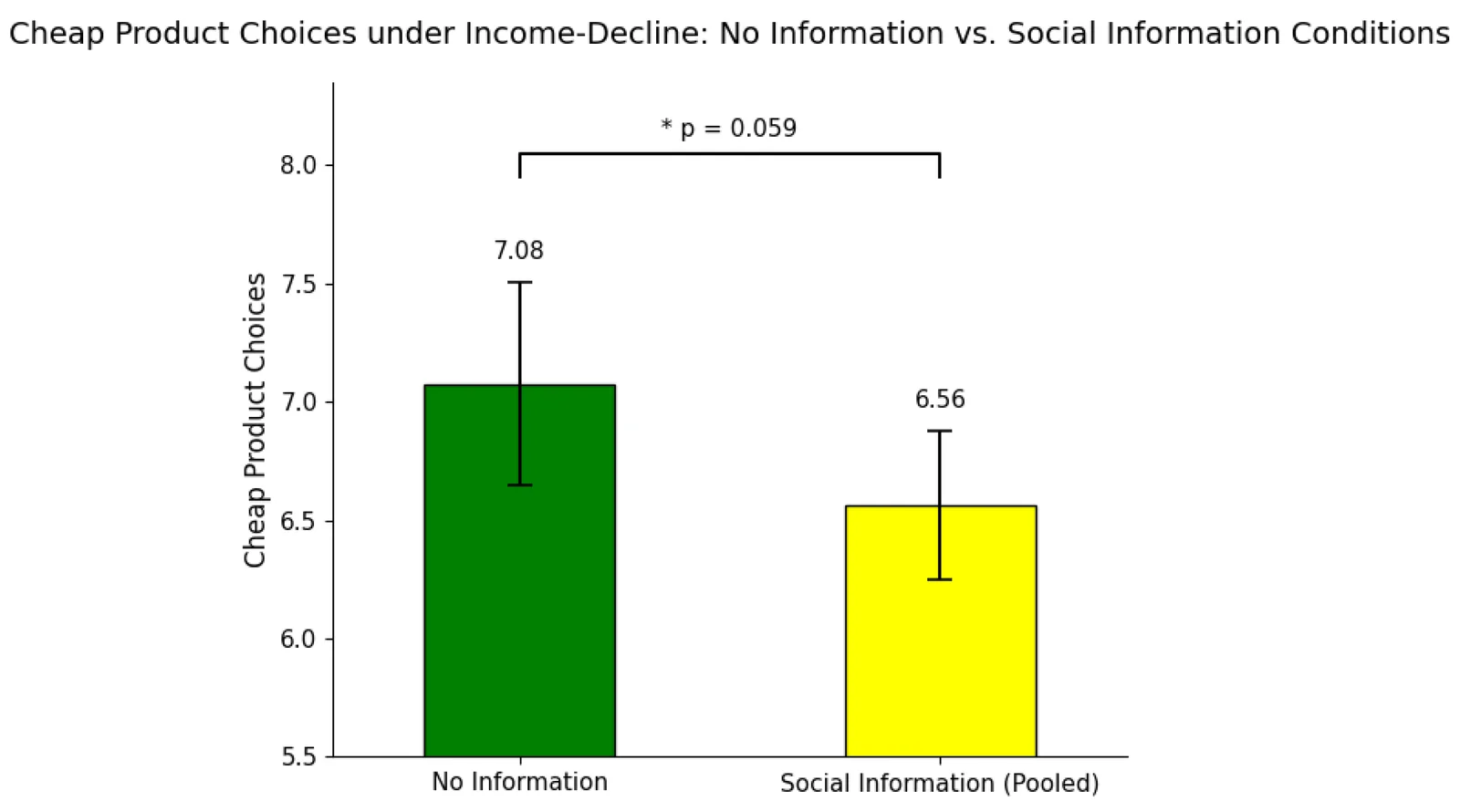
Mean number of cheap product choices under the hypothetical income-decline scenario: the no-information condition versus the pooled social information condition. The pooled condition combines the cheap-salience (cheap-majority) and expensive-salience (expensive-minority) conditions within the nine-product analytical sample with Q9 excluded. Results are based on an independent-samples *t*-test using participant-level choice totals. Error bars represent 95% confidence intervals.

**Figure A5:**
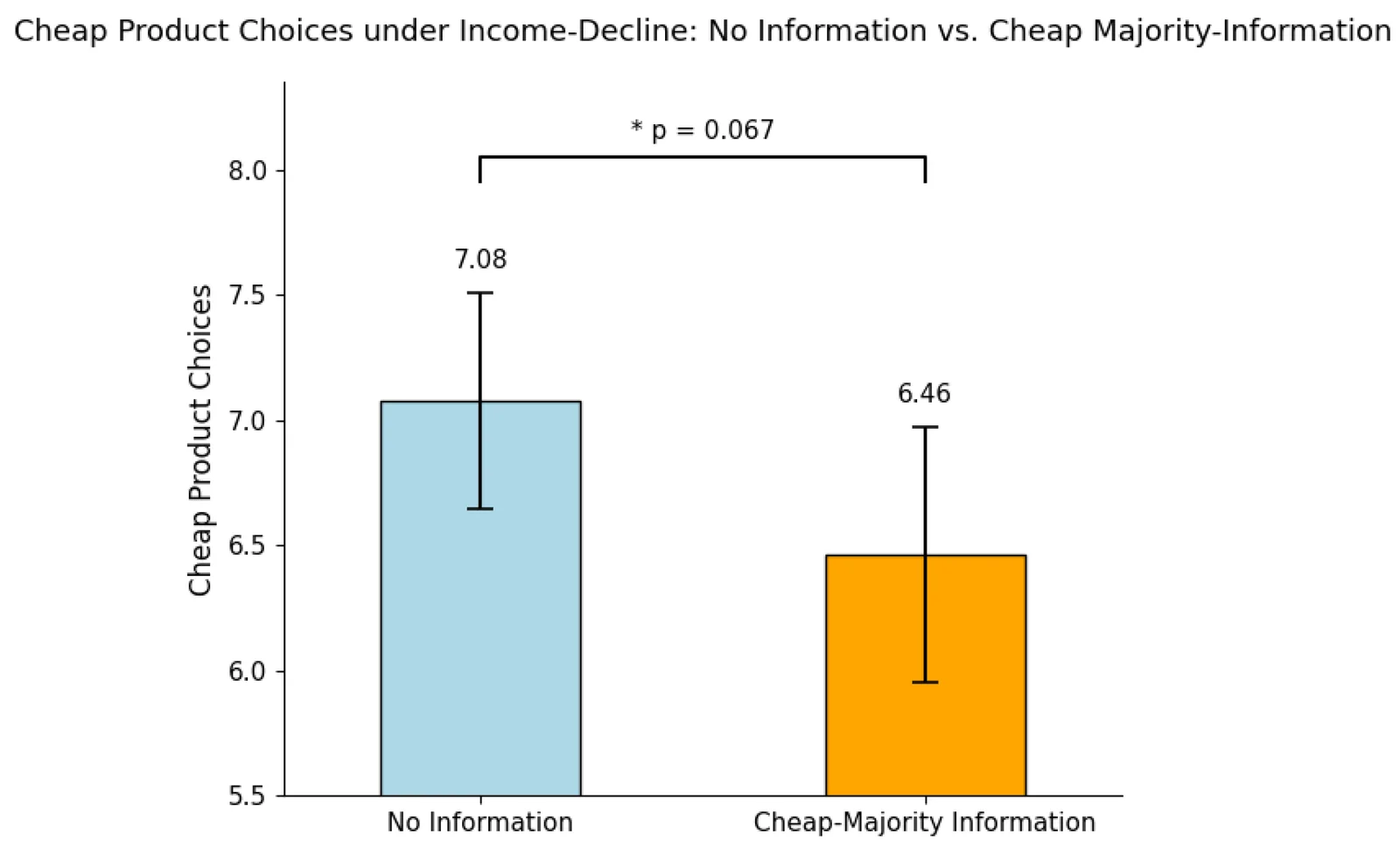
Mean number of cheap product choices under the hypothetical income-decline scenario: the no-information condition versus the cheap-salience (cheap-majority) condition within the nine-product analytical sample with Q9 excluded. Results are based on an independent-samples *t*-test using participant-level choice totals. Error bars represent 95% confidence intervals.

**Figure A6:**
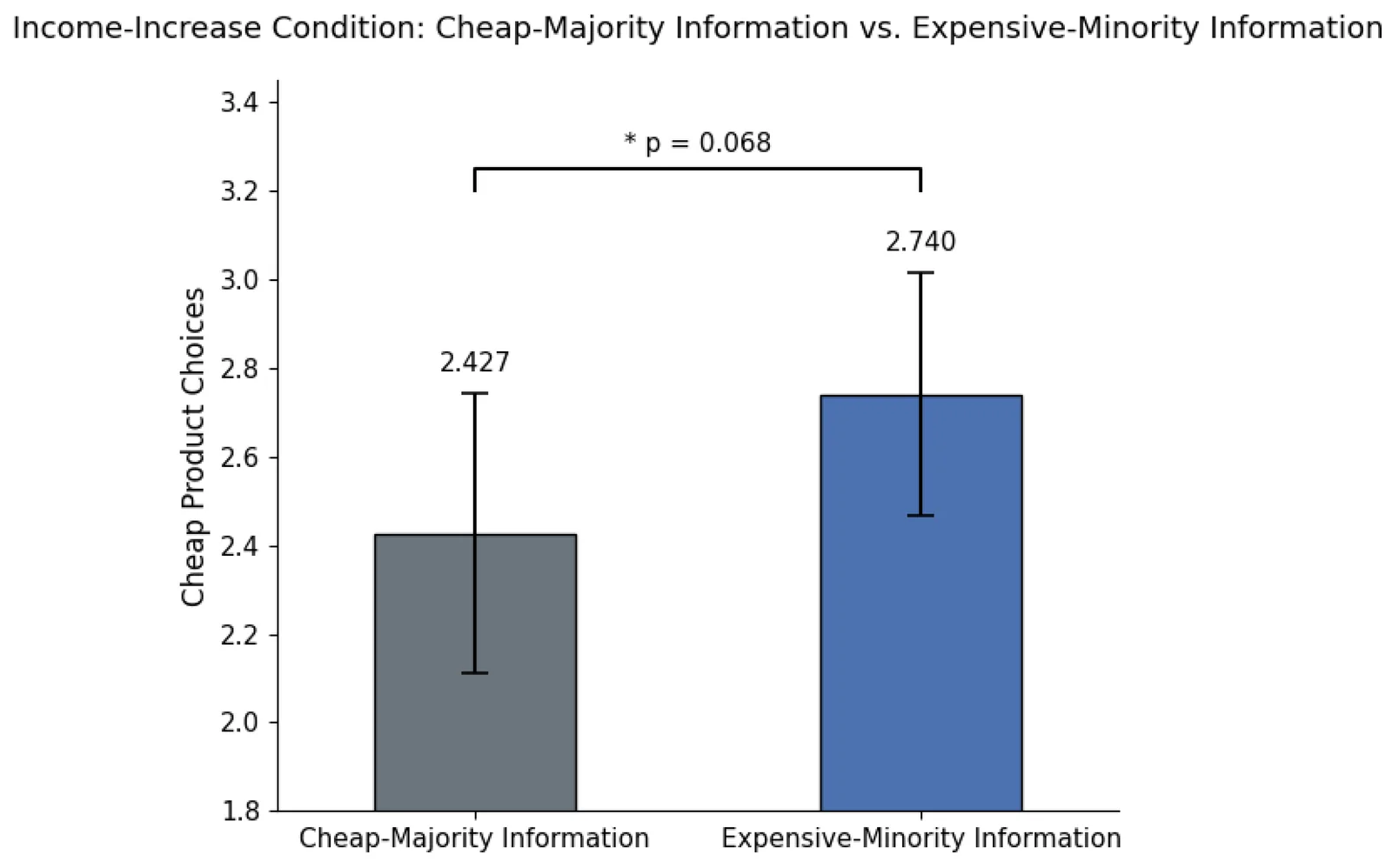
Mean number of cheap product choices under the hypothetical income-increase scenario: the cheap-salience (cheap-majority) condition versus the expensive-salience (expensive-minority) condition, restricted to Q3, Q4, Q8, and Q12. Results are based on an independent-samples *t*-test using participant-level choice totals. Given the directional nature of Hypothesis 6, the one-tailed *p*-value is reported. Error bars represent 95% confidence intervals.

**Figure A7:**
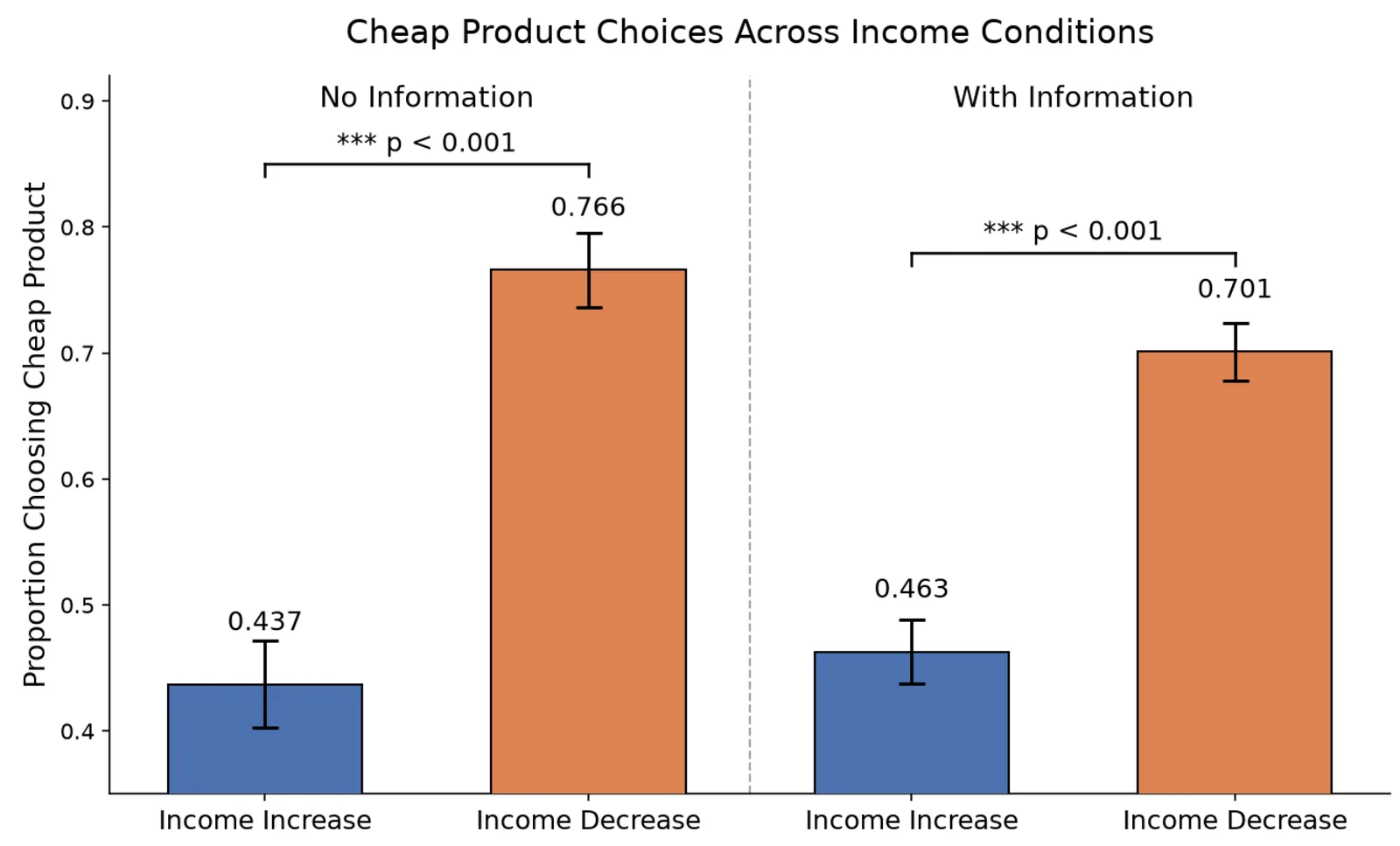
Proportion of cheap product choices by hypothetical income trajectory after excluding all participants with repeated IDs. The left panel compares the income-increase and income-decrease scenarios in the no-information condition. The right panel presents the corresponding comparison in the pooled social information condition, which combines the cheap-salience and expensive-salience conditions. Error bars represent 95% confidence intervals.

**Figure A8:**
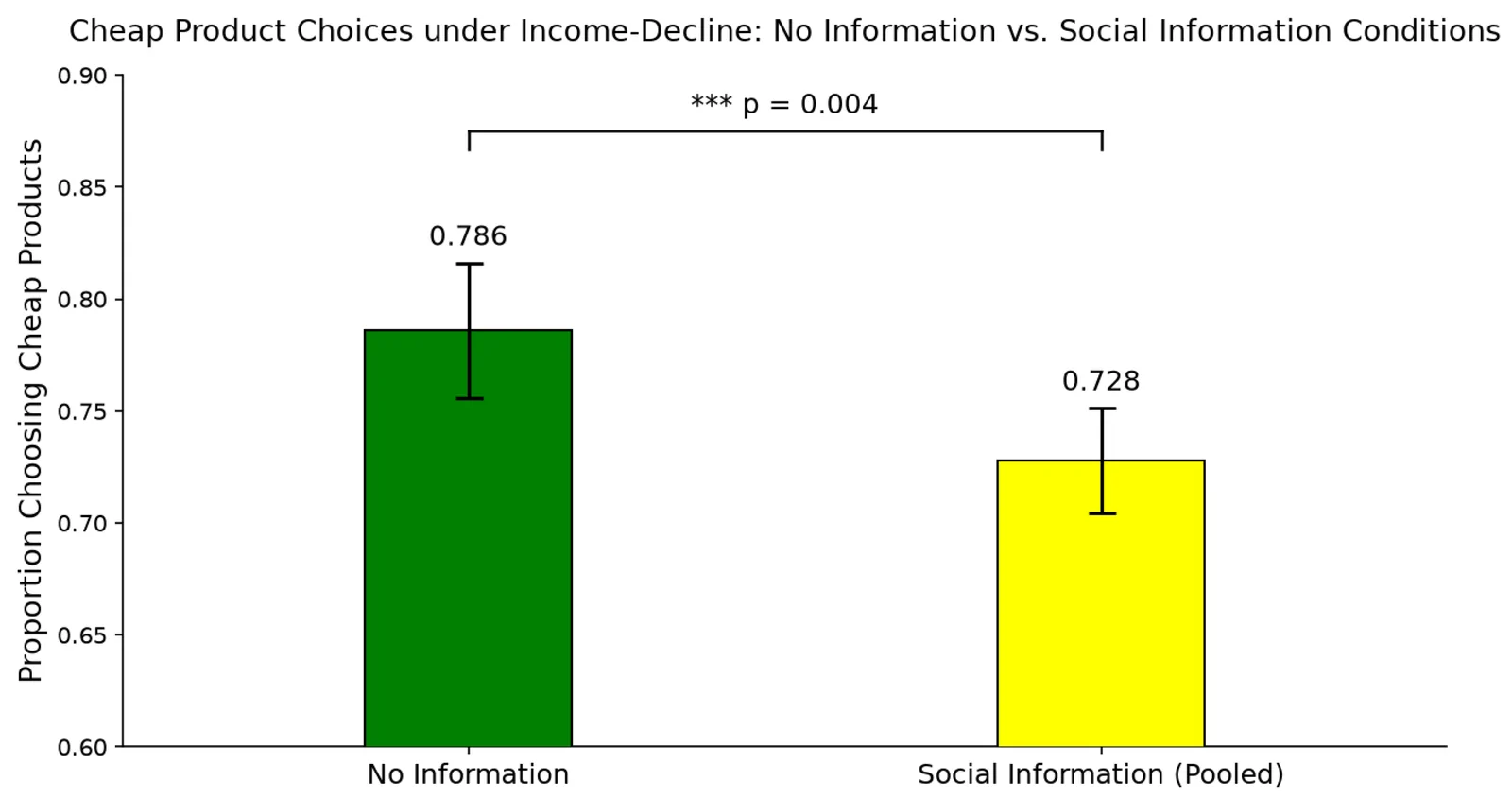
Proportion of cheap product choices under the hypothetical income-decrease scenario after excluding all participants with repeated IDs: the no-information condition versus pooled social information indicating an underlying cheap majority within the nine-product analytical sample. Error bars represent 95% confidence intervals.

**Figure A9:**
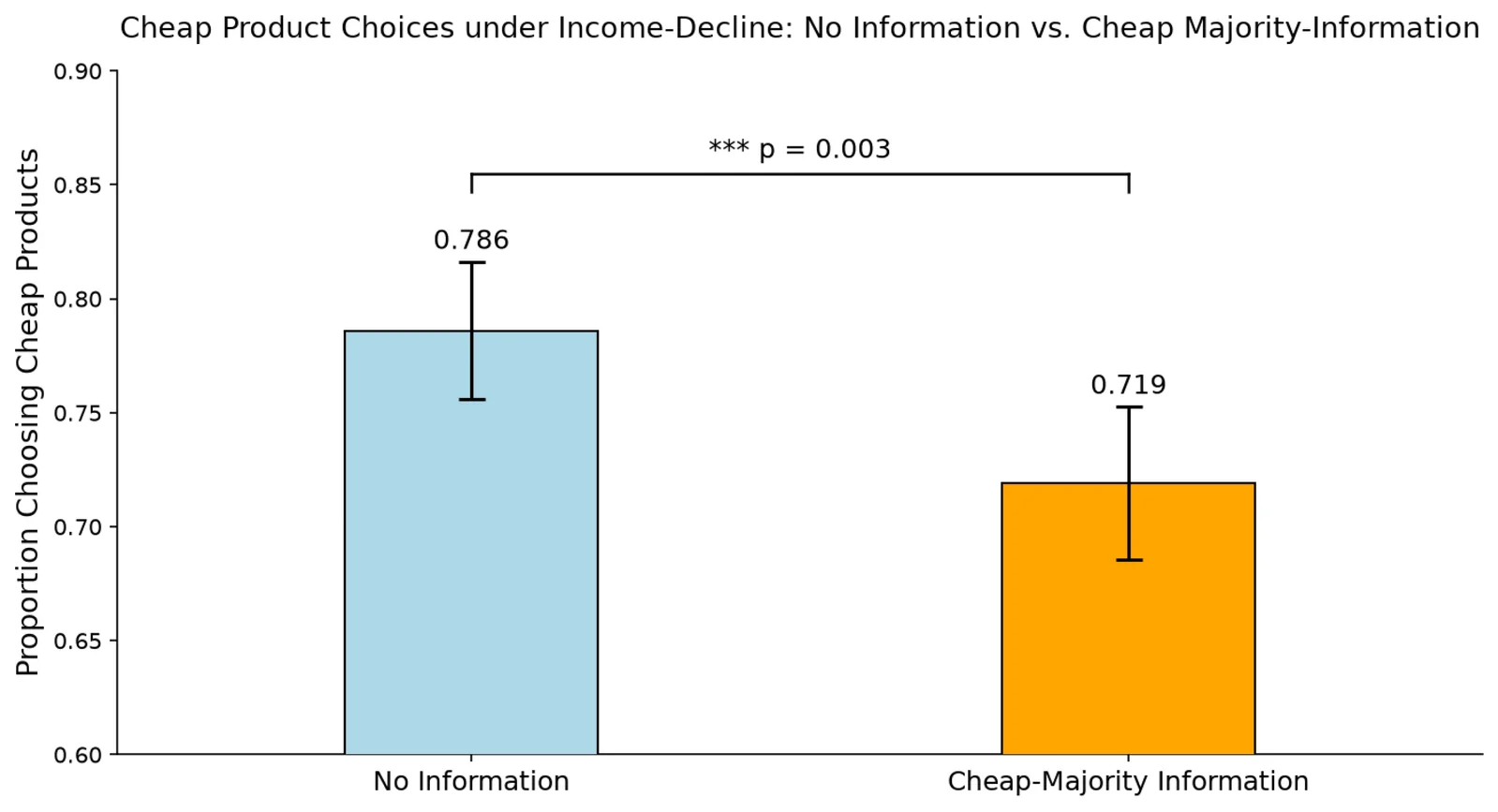
Proportion of cheap product choices under the hypothetical income-decrease scenario after excluding all participants with repeated IDs: the no-information condition versus the cheap-salience condition within the nine-product analytical sample. Error bars represent 95% confidence intervals.

**Figure A10:**
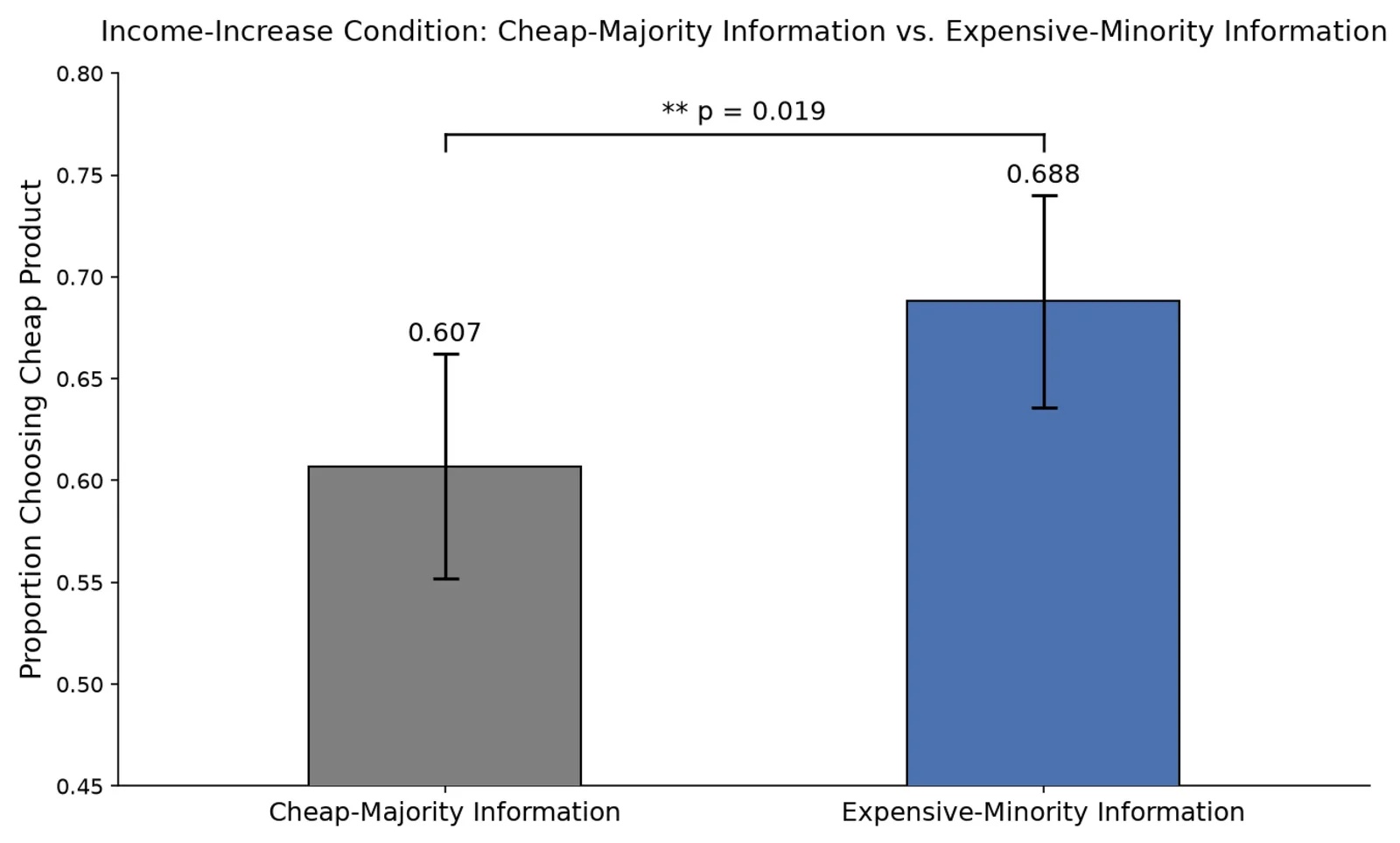
Proportion of cheap product choices under the hypothetical income-increase scenario after excluding all participants with repeated IDs: the cheap-salience (cheap-majority) condition versus the expensive-salience (expensive-minority) condition, restricted to Q3, Q4, Q8, and Q12. Given the directional nature of Hypothesis 6, the one-tailed *p*-value is reported. Error bars represent 95% confidence intervals.

## Data Availability

The original contributions presented in this study are included in the article/Supplementary Materials. Further inquiries can be directed to the corresponding authors.

## Supplementary Materials

The following supporting information can be downloaded at https://www.mdpi.com/article/10.3390/bs16091648/s1.

## Appendix A. Additional Tables and Analysis

**Table A1:** Summary table of variables (part 1).

Variable	Definition	Coding
Conformity	Indicator for whether the participant’s choice conforms to the majority option presented in the social information treatment	Binary variable if the participant chooses the majority option (cheap or expensive, depending on treatment) = 1, otherwise = 0
Income Decrease	Indicator for pessimistic income trajectories treatment	Dummy variable: If the participant is assigned to the income decrease condition = 1, and if assigned to the income increase condition = 0
Income Increase	Indicator for optimistic income trajectories treatment	Dummy variable: If the participant is assigned to the income increase condition = 0, and if assigned to the income decrease condition = 1
Cheap Salience	Indicator for given the cheap information	Dummy variable: If given the cheap products’ information = 1 (C1, C2), and given the expensive products’ information = 0 (c11, c22)
Expensive Salience	Indicator for given the expensive information	Dummy variable: If given the expensive products’ information = 0 (c11, c22), and given the cheap products’ information = 1 (C1, C2)
Income Decrease × Cheap Salience	Interaction between pessimistic income trajectories and cheap-majority	Product of Income Decrease and Cheap Salience

**Table A2:** Summary table of variables (part 2).

Variable	Definition	Coding
Gender	Gender indicator	Dummy variable equal to 1 if female, and 0 if male
Age	Age group indicator	Dummy variable equal to 1 if age is between 18 and 25, and 2 if age is between 26 and 30, and 3 if age is between 31 and 35, and 4 if age is between 36 and 40, and 5 if age is above 40.
Income	Monthly income category	Dummy variable equal to 1 if monthly income is below 2000, and 2 if monthly income is between 2001 and 4000, and 3 if monthly income is between 4001 and 6000, and 4 if monthly income is between 6001 and 8000, and 5 if monthly income is between 8001 and 10,000, and 6 if monthly income is between 10,001 and 15,000, and 7 if monthly income is between 15,001 and 20,000, and 8 if monthly income is between 20,001 and 25,000, and 9 if monthly income is above 25,000
Residence	Residential location	Dummy variable equal to 1 if the participant lives in rural, and 2 if the participant lives in town, and 3 if the participant lives in city.
Growth perception	Economic growth perception	Dummy variable equal to 1 if the participant reports very pessimistic, and 2 if pessimistic, and 3 if slightly pessimistic, and 4 if neutral, and 5 if slightly optimistic, and 6 if optimistic, and 7 if very optimistic.
Education	Education level	Dummy variable equal to 1 if vocational school, and 2 if college, and 3 if bachelor, and 4 if master or Ph.D.

**Table A3:** Participant-level Tobit regression—robustness check for Table 3.

VARIABLES	(1)	(2)	(3)	(4)	(5)
	Cheap Choice	Cheap Choice	Cheap Choice	Cheap Choice	Cheap Choice
Cheap Majority Information	−0.779 *	−0.748 *	−0.921 **	−0.829 *	−0.885 *
	(0.454)	(0.451)	(0.451)	(0.447)	(0.452)
Gender: Female		−0.761	−0.514	−0.447	−0.480
		(0.527)	(0.536)	(0.545)	(0.543)
Age: 26–30			0.855	1.012	1.036
			(0.703)	(0.822)	(0.789)
Age: 31–35			0.647	1.155	1.214 *
			(0.603)	(0.742)	(0.704)
Age: 36–40			−0.781	−0.336	0.001
			(0.666)	(0.774)	(0.751)
Age: >40			−0.282	0.076	0.381
			(0.781)	(0.855)	(0.852)
Income: 2001–4000				−0.083	0.041
				(0.956)	(0.935)
Income: 4001–6000				−0.824	−0.061
				(0.970)	(0.944)
Income: 6001–8000				0.167	0.598
				(0.938)	(0.918)
Income: 8001–10,000				−0.142	0.249
				(0.992)	(0.971)
Income: 10,001–15,000				−0.815	−0.328
				(1.015)	(0.990)
Income: 15,001–20,000				−0.652	−0.096
				(1.111)	(1.099)
Income: 20,001–25,000				−2.138	−1.647
				(1.308)	(1.307)
Income: >25,000				−1.984	−1.030
				(1.639)	(1.590)
Residence: Town				−0.751	−1.482
				(1.418)	(1.372)
Residence: City				−0.676	−1.706
				(1.336)	(1.315)
Economic Outlook: Very Pessimistic					3.521 **
					(1.613)
Economic Outlook: Pessimistic					−0.196
					(0.815)
Economic Outlook: Slightly Pessimistic					−0.362
					(0.648)
Economic Outlook: Slightly Optimistic					−0.587
					(0.740)
Economic Outlook: Optimistic					−2.275 **
					(1.079)
Economic Outlook: Very Optimistic					−3.532 ***
					(1.300)
Education: College					−1.265
					(1.701)
Education: Bachelor					−1.168
					(1.620)
Education: Master/Ph.D.					−0.640
					(1.735)
Constant	7.621 ***	8.176 ***	7.936 ***	8.657 ***	10.635 ***
	(0.327)	(0.507)	(0.613)	(1.310)	(2.115)
Observations	157	157	157	157	157
Log-likelihood	−314.85	−313.81	−310.87	−307.16	−296.99
AIC	635.70	635.62	637.75	650.32	647.97
McFadden $R^{2}$	0.005	0.008	0.017	0.029	0.061

Standard errors in parentheses; *** *p* < 0.01, ** *p* < 0.05, * *p* < 0.1. Note: Participant-level two-limit Tobit models (lower bound 0, upper bound 9), corresponding to Table 3. Each observation represents one participant (N = 157: 79 in Treatment A2, 78 in Treatment C2), with the dependent variable being the total number of cheap products chosen out of 9 eligible product pairs. This specification addresses the non-independence of repeated observations by aggregating to the participant level, eliminating within-person correlation. Results are substantively identical to Table 3, confirming the robustness of the counter-conformity effect.

**Table A4:** Panel logistic regression (random effects)—robustness check for Table 4.

VARIABLES	(1)	(2)	(3)	(4)	(5)
	Conformity	Conformity	Conformity	Conformity	Conformity
Income Decrease	0.267 ***	0.275 ***	0.258 ***	0.236 ***	0.277 ***
	(0.088)	(0.088)	(0.089)	(0.086)	(0.090)
Gender: Female		−0.121	−0.120	−0.094	−0.069
		(0.100)	(0.102)	(0.099)	(0.099)
Age: 26–30			0.130	0.236 *	0.244 *
			(0.128)	(0.135)	(0.134)
Age: 31–35			0.100	0.252 *	0.257 *
			(0.113)	(0.133)	(0.134)
Age: 36–40			0.045	0.138	0.107
			(0.161)	(0.171)	(0.171)
Age: >40			−0.144	−0.004	0.028
			(0.154)	(0.161)	(0.172)
Income: 2001–4000				−0.306 *	−0.347 **
				(0.176)	(0.174)
Income: 4001–6000				−0.144	−0.159
				(0.181)	(0.179)
Income: 6001–8000				−0.070	−0.060
				(0.183)	(0.182)
Income: 8001–10,000				−0.121	−0.126
				(0.198)	(0.196)
Income: 10,001–15,000				−0.570 ***	−0.569 ***
				(0.186)	(0.188)
Income: 15,001–20,000				−0.235	−0.306
				(0.227)	(0.230)
Income: 20,001–25,000				−0.604 **	−0.651 **
				(0.258)	(0.268)
Income: >25,000				−0.783 **	−0.710 **
				(0.319)	(0.320)
Residence: Town				−0.239	−0.143
				(0.268)	(0.269)
Residence: City				0.102	0.157
				(0.258)	(0.259)
Economic Outlook: Very Pessimistic					−0.424
					(0.276)
Economic Outlook: Pessimistic					−0.231
					(0.165)
Economic Outlook: Slightly Pessimistic					−0.227 *
					(0.125)
Economic Outlook: Slightly Optimistic					−0.232
					(0.142)
Economic Outlook: Optimistic					−0.067
					(0.171)
Economic Outlook: Very Optimistic					−0.868 ***
					(0.269)
Education: College					0.003
					(0.260)
Education: Bachelor					−0.061
					(0.248)
Education: Master/Ph.D.					0.037
					(0.267)
Constant	0.657 ***	0.742 ***	0.704 ***	0.825 ***	0.957 ***
	(0.062)	(0.094)	(0.117)	(0.280)	(0.339)
Observations	3090	3090	3090	3090	3090
Number of Participants	309	309	309	309	309
Observations	3090	3090	3090	3090	3090
Number of Participants	309	309	309	309	309
Log-likelihood	−1920.85	−1920.12	−1918.38	−1904.34	−1897.52
AIC	3847.69	3848.23	3852.75	3844.68	3849.03
McFadden $R^{2}$	0.002	0.003	0.004	0.011	0.014

Standard errors in parentheses; *** *p* < 0.01, ** *p* < 0.05, * *p* < 0.1. Note: This table presents the robustness check for Table 4 using a panel random-effects logistic regression model (xtlogit) to explicitly account for the nested structure of 10 choices within 309 unique participants. The total number of question-level observations is 3090. The results remain substantively identical to the baseline model, reinforcing the validity of our main findings. Reference categories are identical to Table 4.

**Table A5:** Robustness check—alternative age specifications.

VARIABLES	(1)	(2)	(3)
	Conformity	Conformity	Conformity
Income Decrease	0.209 *	0.229 **	0.216 *
	(0.117)	(0.116)	(0.116)
Cheap Salience	−0.153	−0.156	−0.144
	(0.101)	(0.101)	(0.101)
Income Decrease × Cheap Salience	0.123	0.0999	0.110
	(0.173)	(0.173)	(0.172)
Age (continuous)		0.000123	0.111 **
		(0.00710)	(0.0500)
${\mathrm{Age}}^{2}$			−0.00170 **
			(0.000763)
N	3090	3090	3090

Standard errors in parentheses; * *p* < 0.10, ** *p* < 0.05. Note: This table presents robustness checks using alternative specifications for the age variable. Column (1) presents the baseline model with the main interaction terms. Column (2) introduces age as a linear continuous variable (derived from cohort midpoints). Column (3) includes a quadratic term (${\mathrm{Age}}^{2}$). The highly significant positive coefficient of Age and negative coefficient of ${\mathrm{Age}}^{2}$ explicitly show a quadratic (inverted U-shaped) relationship between age and consumer conformity, while our core baseline results remain robust and unchanged.

**Table A6:** Robustness check—alternative income specifications.

VARIABLES	(1)	(2)	(3)
	Conformity	Conformity	Conformity
Income Decrease	0.209 *	0.188 *	0.200 *
	(0.117)	(0.113)	(0.114)
Cheap Salience	−0.153	−0.164	−0.154
	(0.101)	(0.101)	(0.101)
Income Decrease × Cheap Salience	0.123	0.170	0.162
	(0.173)	(0.175)	(0.175)
Log monthly income		−0.152 **	0.744
		(0.0606)	(0.752)
Log monthly ${\mathrm{income}}^{2}$			−0.0533
			(0.0446)
N	3090	3090	3090

Standard errors in parentheses; * *p* < 0.10, ** *p* < 0.05. Note: This table presents robustness checks using alternative continuous specifications for the baseline income variable. Column (1) presents the baseline model for comparison. Column (2) introduces baseline absolute income as a log-transformed continuous variable (*Log monthly income*), showing a significant linear negative effect on consumer conformity. Column (3) introduces a quadratic term (*Log monthly ${\mathrm{income}}^{2}$*) to test for non-linearities, which yields non-significant coefficients.

**Table A7:** Participant-level Tobit regression using the full 10-product sample: Robustness check for Table 3.

VARIABLES	(1)	(2)	(3)	(4)	(5)
	Cheap Choice	Cheap Choice	Cheap Choice	Cheap Choice	Cheap Choice
Cheap Salience	−1.003 **	−0.967 **	−1.163 **	−1.048 **	−1.155 **
	(0.483)	(0.479)	(0.479)	(0.474)	(0.479)
Gender: Female		−0.772	−0.517	−0.455	−0.530
		(0.556)	(0.564)	(0.574)	(0.571)
Age: 26–30			0.827	1.077	1.127
			(0.743)	(0.863)	(0.829)
Age: 31–35			0.751	1.370 *	1.420 *
			(0.639)	(0.783)	(0.742)
Age: 36–40			−0.995	−0.439	−0.054
			(0.708)	(0.817)	(0.794)
Age: >40			−0.352	0.044	0.367
			(0.827)	(0.903)	(0.898)
Income: 2001–4000				−0.009	0.128
				(1.018)	(0.995)
Income: 4001–6000				−0.893	−0.077
				(1.032)	(1.003)
Income: 6001–8000				0.206	0.656
				(0.990)	(0.968)
Income: 8001–10,000				−0.266	0.154
				(1.046)	(1.023)
Income: 10,001–15,000				−1.006	−0.470
				(1.076)	(1.048)
Income: 15,001–20,000				−0.953	−0.299
				(1.172)	(1.159)
Income: 20,001–25,000				−2.306 *	−1.672
				(1.391)	(1.388)
Income: >25,000				−2.292	−1.248
				(1.742)	(1.687)
Residence: Town				−0.200	−1.219
				(1.471)	(1.443)
Residence: City				−0.316	−1.628
				(1.380)	(1.380)
Economic Outlook: Very Pessimistic					3.841 **
					(1.682)
Economic Outlook: Pessimistic					−0.325
					(0.865)
Economic Outlook: Slightly Pessimistic					−0.342
					(0.684)
Economic Outlook: Slightly Optimistic					−0.658
					(0.782)
Economic Outlook: Optimistic					−2.851 **
					(1.137)
Economic Outlook: Very Optimistic					−3.780 ***
					(1.383)
Education: College					−1.307
					(1.806)
Education: Bachelor					−1.286
					(1.721)
Education: Master/Ph.D.					−0.927
					(1.840)
Constant	8.168 ***	8.727 ***	8.515 ***	8.873 ***	11.318 ***
	(0.347)	(0.533)	(0.640)	(1.334)	(2.232)
Observations	157	157	157	157	157

Note: The analysis includes participants in the no-information and cheap-salience conditions under the hypothetical income-decrease scenario. The dependent variable is the participant-level number of cheaper products selected across all 10 product-choice tasks (range: 0–10). A two-limit Tobit model is estimated with lower and upper bounds of 0 and 10, respectively. Because Q9 presented cheap-minority information in the cheap-salience condition, whereas the remaining nine products presented cheap-majority information, the coefficient for Cheap Salience estimates its overall effect relative to no information rather than the effect of cheap-majority information specifically. The reference categories are “No Information,” “Male,” “Age 18–25,” “Income below 2000 yuan,” “Rural Residence,” “Neutral Economic Outlook,” and “Vocational School.” Standard errors are reported in parentheses. *** *p* < 0.01, ** *p* < 0.05, * *p* < 0.10.

**Table A8:** Effect sizes for formal proportion comparisons reported in the main text.

Comparison	Proportion 1	Proportion 2	Difference	Cohen’s *h*
Anticipated income decrease vs. anticipated income increase: no information	0.766	0.440	32.6 percentage points	0.68
Anticipated income decrease vs. anticipated income increase: pooled social information	0.702	0.463	23.9 percentage points	0.49
Pooled social information vs. no information: anticipated income decrease	0.729	0.786	−5.7 percentage points	0.13
Cheap-majority information vs. no information: anticipated income decrease	0.718	0.786	−6.8 percentage points	0.16
Expensive-minority vs. cheap-majority information: anticipated income increase	0.685	0.607	7.8 percentage points	0.16

Note: This table reports standardized effect sizes for the formal proportion comparisons presented in the main text. Cohen’s *h* is calculated as $2\arcsin\left(\sqrt{p_{1}}\right)-2\arcsin\left(\sqrt{p_{2}}\right)$. The sign of Cohen’s *h* and the percentage-point difference indicates the direction of the comparison, whereas the absolute value indicates its standardized magnitude. Conventional benchmarks of |h|=0.20, 0.50, and 0.80 correspond to small, medium, and large effects, respectively. Percentage-point differences are reported to facilitate interpretation in the original outcome metric.

**Table A9:** Benjamini–Hochberg adjustment for primary and theoretically central tests reported in the main text.

Test	Unadjusted *p*-Value	BH-FDR-Adjusted *p*-Value	Adjusted Result
Anticipated income decrease vs. anticipated income increase: no-information conditions	<0.001	<0.001	Significant
Anticipated income decrease vs. anticipated income increase: pooled social information conditions	<0.001	<0.001	Significant
Cheap-majority information vs. no information: anticipated income decrease	0.0030	0.0074	Significant
Pooled social information vs. no information: anticipated income decrease	0.0041	0.0082	Significant
Expensive-minority information vs. cheap-majority information: anticipated income increase	0.0216	0.0360	Significant
Cheap Salience coefficient: Table 5, Model 1	0.0444	0.0634	Marginal significant
Income Decrease coefficient: Table 5, Model 1	0.0886	0.1108	Not significant
Income Decrease × Cheap Salience: Table 5, Model 1	0.4318	0.4318	Not significant

Note: The Benjamini–Hochberg procedure was applied jointly to the primary tests reported in the main text. Directional alternatives concerning the same underlying comparison were treated as a single test, and regression coefficients and corresponding marginal effects representing the same effect were not counted separately. Accordingly, the Tobit estimates and average marginal effects reported in Table 3 were not included as additional tests, because they correspond to the same cheap-majority-information versus no-information comparison already represented above. Because Hypothesis 6 specified a directional prediction, its one-tailed *p*-value was included in the adjustment (unadjusted *p* = 0.0216, BH-FDR-adjusted *p* = 0.0360).

**Table A10:** Comparison of pilot and no-information choice proportions.

Product Option	Income Increase: No Information	Income Increase: Pilot	Income Decrease: No Information	Income Decrease: Pilot
Luckin coffee	62.50%	66.7%	97.47%	100.0%
Standard baseball cap	55.00%	60.0%	84.81%	86.7%
Standard shoes	42.50%	46.7%	82.28%	76.7%
Boxed meal	28.75%	26.7%	55.70%	56.7%
Loose strawberries (25 pieces)	52.50%	43.3%	82.28%	83.3%
Basic badminton racket	61.25%	66.7%	83.54%	86.7%
Practical water bottle	21.25%	16.7%	58.23%	46.7%
Basic shampoo (400 mL)	25.00%	23.3%	65.82%	63.3%
Basic skincare product (75 mL)	25.00%	33.3%	69.62%	70.0%
Higher-specification model from an affordable brand	66.25%	70.0%	86.08%	86.7%

Note: Entries report the percentage selecting the relatively cheaper option for each product. The pilot samples included 30 participants in each income-trajectory scenario. The corresponding no-information conditions included 80 participants under anticipated income increase and 79 participants under anticipated income decline. Across the 10 products, the pilot and no-information proportions were correlated at r=0.964 under anticipated income increase and r=0.971 under anticipated income decline. The corresponding mean absolute differences were 4.84 and 3.02 percentage points. These comparisons are descriptive and should not be interpreted as equivalence tests or as evidence that the pilot proportions precisely estimate population preferences.

## Appendix B. Robustness Analysis Excluding All Participants with Repeated IDs

As described in the main text, nine unique participant IDs were associated with responses in more than one experimental condition because of platform assignment anomalies. Six IDs appeared in two conditions and three appeared in three conditions, producing 21 response records in total. For the main analysis, we retained only the first completed response associated with each ID and excluded the 12 subsequent duplicate records. This procedure yielded the main analytical sample of 468 participants.

As a robustness check, we adopted a more conservative exclusion rule and removed all records associated with these nine IDs, including their first completed responses. For four of the nine IDs, the first completed response belonged to experimental conditions outside the scope of the present study, such as conditions involving incentive-related manipulations. Their subsequent responses in the six conditions examined in this paper had already been removed as duplicate records when constructing the main analytical sample. Consequently, these four IDs contributed no retained observations to the 468-participant sample. The first completed responses of the remaining five IDs were included in the main analytical sample and were removed for the present robustness analysis. This resulted in a reduced sample of 463 participants.

The condition-specific sample sizes were 79 in the income-increase/no-information condition, 79 in the income-decrease/no-information condition, 75 in the income-increase/cheap-salience condition, 77 in the income-decrease/cheap-salience condition, 76 in the income-increase/expensive-salience condition, and 77 in the income-decrease/expensive-salience condition. We repeated the principal analyses reported in the main text using the same variable definitions, product restrictions, and statistical procedures. The results are presented below in the same order as the corresponding main-text analyses.

Overall, excluding all observations associated with the nine repeated IDs did not substantively alter the findings. The estimated differences remained similar in direction and magnitude to those reported in the main text, and the substantive conclusions were unchanged.

**Table A11:** Tobit regression results after excluding all participants with repeated IDs of Table 3.

VARIABLES	(1)	(2)	(3)	(4)	(5)
	Cheap Choice	Cheap Choice	Cheap Choice	Cheap Choice	Cheap Choice
Cheap-Majority Information	−0.76805 *	−0.73984	−0.91926 **	−0.81481 *	−0.85673 **
	(0.459)	(0.457)	(0.449)	(0.441)	(0.432)
Gender: Female		−0.75636	−0.51202	−0.43536	−0.40857
		(0.525)	(0.548)	(0.545)	(0.527)
Age: 26–30			0.85600	1.03652	1.09407
			(0.672)	(0.867)	(0.866)
Age: 31–35			0.64814	1.18262	1.28178
			(0.626)	(0.796)	(0.787)
Age: 36–40			−0.78278	−0.31867	0.04016
			(0.637)	(0.757)	(0.737)
Age: >40			−0.26653	0.15077	0.49674
			(0.847)	(0.926)	(0.854)
Income: 2001–4000				−0.03455	0.14691
				(0.777)	(0.692)
Income: 4001–6000				−0.84425	−0.08347
				(0.930)	(0.908)
Income: 6001–8000				0.13999	0.54803
				(0.850)	(0.805)
Income: 8001–10,000				−0.16464	0.22525
				(0.963)	(0.947)
Income: 10,001–15,000				−0.84509	−0.36895
				(0.909)	(0.896)
Income: 15,001–20,000				−0.68454	−0.14085
				(1.171)	(1.115)
Income: 20,001–25,000				−2.17015	−1.67712
				(1.321)	(1.203)
Income: >25,000				−2.03431	−1.37942
				(1.667)	(1.602)
Residence: Town				−0.78472	−1.61660
				(1.358)	(1.304)
Residence: City				−0.68700	−1.75280
				(1.252)	(1.224)
Economic Outlook: Very Pessimistic					3.54140 **
					(1.670)
Economic Outlook: Pessimistic					−0.10450
					(0.740)
Economic Outlook: Slightly Pessimistic					−0.36650
					(0.616)
Economic Outlook: Slightly Optimistic					−0.56932
					(0.679)
Economic Outlook: Optimistic					−2.26200 *
					(1.284)
Economic Outlook: Very Optimistic					−3.69261 ***
					(0.923)
Education: College					−2.31283
					(1.633)
Education: Bachelor					−2.22154
					(1.553)
Education: Master/Ph.D.					−1.70928
					(1.657)
Constant	7.62502 ***	8.17661 ***	7.93626 ***	8.65926 ***	11.65447 ***
	(0.324)	(0.488)	(0.598)	(1.231)	(2.027)
Observations	1404	1404	1404	1404	1404

Note: Robust standard errors are reported in parentheses. The analysis excludes all observations associated with the nine repeated participant IDs. The sample is restricted to the hypothetical income-decrease scenario and compares the no-information condition with the cheap-salience condition within the nine-product analytical sample, with Q9 excluded. The reference categories are “No Information,” “Male,” “Age 18–25,” “Income below 2000 yuan,” “Rural Residence,” “Neutral Economic Outlook,” and “Vocational School.” *** *p* < 0.01, ** *p* < 0.05, and * *p* < 0.10.

**Table A12:** Logistic regression results after excluding all participants with repeated IDs of Table 4.

VARIABLES	(1)	(2)	(3)	(4)	(5)
	Conformity	Conformity	Conformity	Conformity	Conformity
Income Decrease	0.253 ***	0.261 ***	0.243 ***	0.231 ***	0.275 ***
	(0.088)	(0.088)	(0.090)	(0.090)	(0.090)
Gender: Female		−0.103	−0.103	−0.083	−0.057
		(0.104)	(0.107)	(0.104)	(0.103)
Age: 26–30			0.127	0.224 *	0.232 *
			(0.123)	(0.128)	(0.126)
Age: 31–35			0.089	0.226 *	0.230 *
			(0.110)	(0.128)	(0.130)
Age: 36–40			0.040	0.121	0.091
			(0.166)	(0.176)	(0.182)
Age: >40			−0.148	−0.017	0.013
			(0.162)	(0.165)	(0.176)
Income: 2001–4000				−0.299 *	−0.343 **
				(0.163)	(0.162)
Income: 4001–6000				−0.133	−0.148
				(0.178)	(0.177)
Income: 6001–8000				−0.055	−0.046
				(0.165)	(0.168)
Income: 8001–10,000				−0.107	−0.112
				(0.182)	(0.185)
Income: 10,001–15,000				−0.553 ***	−0.556 ***
				(0.175)	(0.176)
Income: 15,001–20,000				−0.219	−0.291
				(0.237)	(0.240)
Income: 20,001–25,000				−0.576 **	−0.630 **
				(0.255)	(0.255)
Income: >25,000				−0.757 **	−0.691 **
				(0.313)	(0.285)
Residence: Town				−0.239	−0.143
				(0.206)	(0.207)
Residence: City				0.102	0.159
				(0.196)	(0.199)
Economic Outlook: Very Pessimistic					−0.430
					(0.297)
Economic Outlook: Pessimistic					−0.227
					(0.163)
Economic Outlook: Slightly Pessimistic					−0.229 *
					(0.125)
Economic Outlook: Slightly Optimistic					−0.228
					(0.142)
Economic Outlook: Optimistic					−0.064
					(0.159)
Economic Outlook: Very Optimistic					−0.857 ***
					(0.228)
Education: College					0.007
					(0.280)
Education: Bachelor					−0.061
					(0.272)
Education: Master/Ph.D.					0.042
					(0.288)
Constant	0.641 ***	0.713 ***	0.683 ***	0.806 ***	0.940 ***
	(0.050)	(0.093)	(0.117)	(0.222)	(0.332)
Observations	3050	3050	3050	3050	3050
Number of Participants	305	305	305	305	305

Note: The sample includes all social information conditions after excluding all observations associated with the nine repeated participant IDs. The reduced sample consists of 305 unique participants making 10 product choices each, yielding 3050 question-level observations. The dependent variable is observed conformity, defined as choosing the option selected by the majority of the corresponding pilot participants. Robust standard errors are reported in parentheses. The reference categories are “Income Increase,” “Male,” “Age 18–25,” “Income below 2000 yuan,” “Rural Residence,” “Neutral Economic Outlook,” and “Vocational School.” *** p<0.01, ** p<0.05, and * p<0.10.

**Table A13:** Panel logistic regression after excluding all participants with repeated IDs of Table 5.

VARIABLES	(1)	(2)	(3)	(4)	(5)
	Conformity	Conformity	Conformity	Conformity	Conformity
Income Decrease	0.180	0.190 *	0.165	0.166	0.211 *
	(0.113)	(0.113)	(0.117)	(0.118)	(0.119)
Cheap Salience	−0.202 **	−0.195 **	−0.196 **	−0.181 *	−0.153
	(0.099)	(0.099)	(0.099)	(0.101)	(0.101)
Income Decrease × Cheap Salience	0.145	0.138	0.153	0.126	0.119
	(0.175)	(0.175)	(0.176)	(0.174)	(0.175)
Gender: Female		−0.094	−0.092	−0.075	−0.050
		(0.103)	(0.107)	(0.104)	(0.103)
Age: 26–30			0.129	0.218 *	0.229 *
			(0.122)	(0.128)	(0.126)
Age: 31–35			0.080	0.205	0.213
			(0.110)	(0.129)	(0.131)
Age: 36–40			0.024	0.092	0.064
			(0.167)	(0.176)	(0.182)
Age: >40			−0.148	−0.032	−0.000
			(0.159)	(0.161)	(0.172)
Income: 2001–4000				−0.309 *	−0.350 **
				(0.163)	(0.162)
Income: 4001–6000				−0.128	−0.146
				(0.179)	(0.178)
Income: 6001–8000				−0.052	−0.049
				(0.170)	(0.175)
Income: 8001–10,000				−0.108	−0.111
				(0.183)	(0.187)
Income: 10,001–15,000				−0.538 ***	−0.544 ***
				(0.175)	(0.177)
Income: 15,001–20,000				−0.181	−0.260
				(0.239)	(0.243)
Income: 20,001–25,000				−0.558 **	−0.615 **
				(0.251)	(0.256)
Income: >25,000				−0.731 **	−0.678 **
				(0.318)	(0.291)
Residence: Town				−0.232	−0.137
				(0.209)	(0.211)
Residence: City				0.116	0.172
				(0.200)	(0.204)
Economic Outlook: Very Pessimistic					−0.425
					(0.301)
Economic Outlook: Pessimistic					−0.221
					(0.164)
Economic Outlook: Slightly Pessimistic					−0.231 *
					(0.124)
Economic Outlook: Slightly Optimistic					−0.220
					(0.144)
Economic Outlook: Optimistic					−0.058
					(0.157)
Economic Outlook: Very Optimistic					−0.833 ***
					(0.227)
Education: College					0.002
					(0.282)
Education: Bachelor					−0.057
					(0.276)
Education: Master/Ph.D.					0.023
					(0.290)
Constant	0.743 ***	0.805 ***	0.778 ***	0.884 ***	1.006 ***
	(0.069)	(0.100)	(0.124)	(0.233)	(0.338)
Observations	3050	3050	3050	3050	3050
Number of Participants	305	305	305	305	305

Note: The sample includes all social information conditions after excluding all observations associated with the nine repeated participant IDs. The reduced sample consists of 305 unique participants making 10 product choices each, yielding 3050 question-level observations. The dependent variable is observed conformity, defined as choosing the option selected by the majority of the corresponding pilot participants. Robust standard errors are reported in parentheses. The reference categories are “Income Increase,” “Expensive Salience,” “Male,” “Age 18–25,” “Income below 2000 yuan,” “Rural Residence,” “Neutral Economic Outlook,” and “Vocational School.” The omitted base-category interaction terms generated by the statistical software are not displayed. *** p<0.01, ** p<0.05, and * p<0.10.

Notes 1 We identified nine unique participant IDs associated with responses in multiple experimental conditions because of platform assignment anomalies. Six IDs appeared in two conditions, while three IDs appeared in three conditions. For each ID, we retained only the first completed response and excluded all subsequent responses, resulting in the removal of 12 duplicate response records and a final analytical sample of 468 participants. The final condition-specific sample sizes were 80 in the income-increase/no-information condition, 79 in the income-decrease/no-information condition, 75 in the income-increase/cheap-salience condition, 78 in the income-decrease/cheap-salience condition, 77 in the income-increase/expensive-salience condition, and 79 in the income-decrease/expensive-salience condition. As a robustness check, we re-estimated the main analyses after excluding all responses associated with these nine IDs. The results were substantively unchanged and are reported in Appendix B. All analyses in the main text are based on the 468-participant sample. 2 The draft time-stamped prepared pre-registration materials are available for transparency at https://osf.io/registries/drafts/67a840efb8f555937f0b1267/review (accessed on 9 February 2025). 3 As a robustness check, we estimated a participant-level Tobit model using all 10 products. Because Q9 presented cheap-minority rather than cheap-majority information, this model estimates the overall effect of cheap salience relative to no information. Results are reported in Appendix A Table A7. 4 Cohen’s *h* provides a standardized measure of differences between proportions. Conventional benchmarks of |h|=0.20, 0.50, and 0.80 correspond to small, medium, and large effects, respectively. Cohen’s *h* values for all formal proportion comparisons are reported in Appendix A Table A8. Benjamini–Hochberg-adjusted *p*-values were also calculated across 10 primary and theoretically central tests. Complete unadjusted and adjusted results are reported in Appendix A Table A9. 5 We also examined this pattern separately for functional and symbolic products. In both categories, participants in the pooled social information conditions selected cheaper products less frequently than those in the no-information condition; see Appendix A Figure A2.
