# Supplementary material for: Challenging the Thrift Paradigm: When Decreasing Income Trajectory Drives Expensive Non-Conformity
Source: Behav Sci (Basel). 2026 Sep 14;16(9):1648. doi: 10.3390/bs16091648 (PMC13603509; doi:10.3390/bs16091648)
Supplement: Supplementary file 1 [file behavsci-16-01648-s001.zip › behavsci-4441219-supplementary.pdf]

Questionnaire

This presents the structure of the questionnaire. The text provided is the actual English translation of the original Chinese questionnaire. While all groups followed the same overall structure, identical sections are presented only once to avoid redundancy across the six groups. The specific experimental interventions for each group, including income scenarios and social information treatments, are clearly labeled and contrasted in the following sections.

Module 1: Introduction and Informed Consent (Common to All Groups)
Thank you for participating in this academic study. Before proceeding, please read the following information carefully.

1. Study Content
This study focuses on consumption behavior. You will be asked to make choices among different consumer products.

2. Duration
Approximately 5 min.

3. Data Confidentiality
All responses will be used for academic research purposes only and **will be kept strictly confidential.**

4. Compensation
You will receive **RMB 1** upon completing the questionnaire, provided that your responses meet the validity requirements described below.

5. Response Requirements
Please read each question carefully and answer truthfully. The questionnaire contains two **attention-check questions**. If the attention-check question is answered incorrectly, the questionnaire will be considered invalid.

6. Informed Consent
By completing and submitting this questionnaire, you acknowledge that you understand the above information and voluntarily agree to participate. You may exit the survey at any time if you do not wish to continue.

Module 2: Income Scenario

Income-Increase Conditions: No Information, Cheap Salience, and Expensive Salience:

(Note: Each income-trajectory scenario included three information conditions: no information, cheap salience, and expensive salience.)

In this survey, you will be asked to make choices under a hypothetical scenario. We will first describe your assumed income situation and then ask about your feelings and decisions under that scenario.

Please imagine that **starting from now, your monthly income continues to increase over a prolonged period**. The following factors may contribute to this sustained income growth:

1. Favorable Economic Environment: The economy is thriving, inflation remains low, and price volatility is limited. Financial markets are relatively stable, and risks associated with investment are modest. The real economy is expanding, supporting a strong labor market. Global trade and supply chains operate smoothly.
2. Positive Industry Outlook: The industry in which you or your income source operates is performing well, with stable demand, limited destructive competition, and a predictable business environment that supports steady income growth.
3. Promising Career Prospects: The projects you are responsible for have strong future potential and a clear career development path, allowing your income to grow steadily.

Together, these factors promote continuous income growth. This helps you better plan your personal finances and career development.

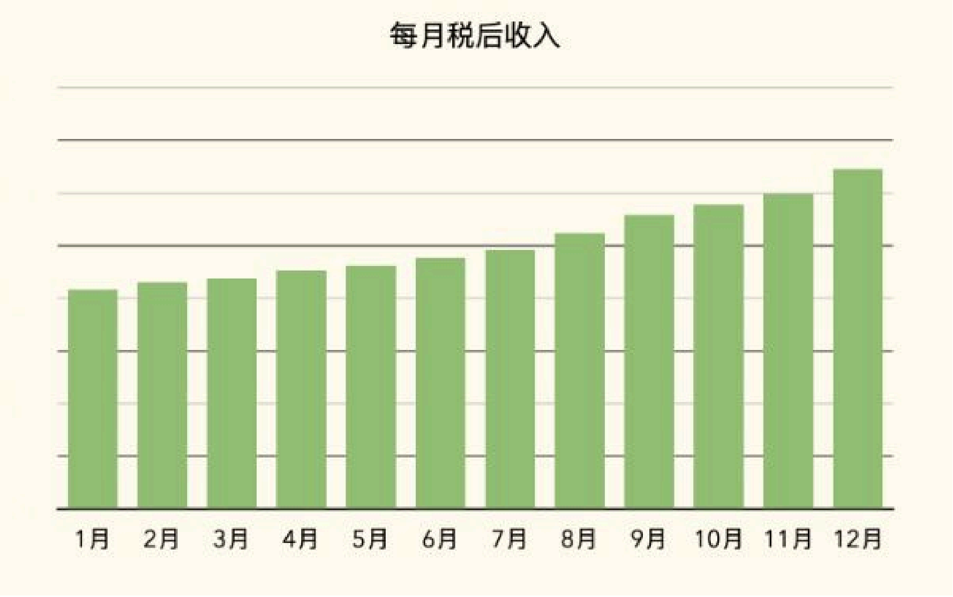

(Note: The Chinese characters "每月税后收入" in the figure translate to "Monthly After-tax Income," and the horizontal axis represents the months from January to December.)

(Note: The following two questions were included as a mental simulation exercise to enhance scenario immersion and to help participants mentally adapt to the hypothetical income scenario before consumption choice tasks.)

Q1. How satisfied are you with your continuously increasing income?
(9-point Likert scale)
1 = Very dissatisfied … 9 = Very satisfied

Very dissatisfied								Very satisfied
1 ○	2 ○	3 ○	4 ○	5 ○	6 ○	7 ○	8 ○	9 ○

Q2. What impacts do you think increasing income has on your life? *(Multiple choices allowed)*

☐ Greater financial security and less concern about future economic fluctuations

☐ Increased planning for investment and financial management

☐ Greater attention to personal health

☐ More opportunities for leisure, travel, and social activities

☐ Improved quality of life and increased consumption of higher-quality products

☐ Regular saving for potential future needs

☐ No noticeable impact

☐ Other: ___________

Income-Decrease Conditions: No Information, Cheap Salience, and Expensive Salience:

(Note: Each income-trajectory scenario included three information conditions: no information, cheap salience, and expensive salience.)

In this survey, you will be asked to make choices under a hypothetical scenario. We will first describe your assumed income situation and then ask about your feelings and decisions under that scenario.
Please imagine that **starting from now, your monthly income continues to decrease over a prolonged period**. The following factors may contribute to this sustained income growth:

1. Adverse Economic Environment: The economy is struggling: growth has slowed, inflation remains elevated, and financial markets are volatile. Investment risks have risen, liquidity is tight, and the labor market is weakening. Global trade tensions and supply chain disruptions further undermine economic stability.
2. Negative Industry Outlook: The industry in which you or your income source operates is under pressure, with falling demand, intense destructive competition, and an unpredictable business climate. Firms face shrinking margins and operational uncertainty, making stable income increasingly difficult to maintain.
3. Uncertain Career Prospects: The projects you are responsible for show limited future potential, and your career trajectory lacks clarity. Opportunities for advancement are scarce, and job security is low. These factors collectively exert downward pressure on your earnings.

Together, these factors ensure continuous income decline. This makes it difficult for you to plan your personal finances and career development.

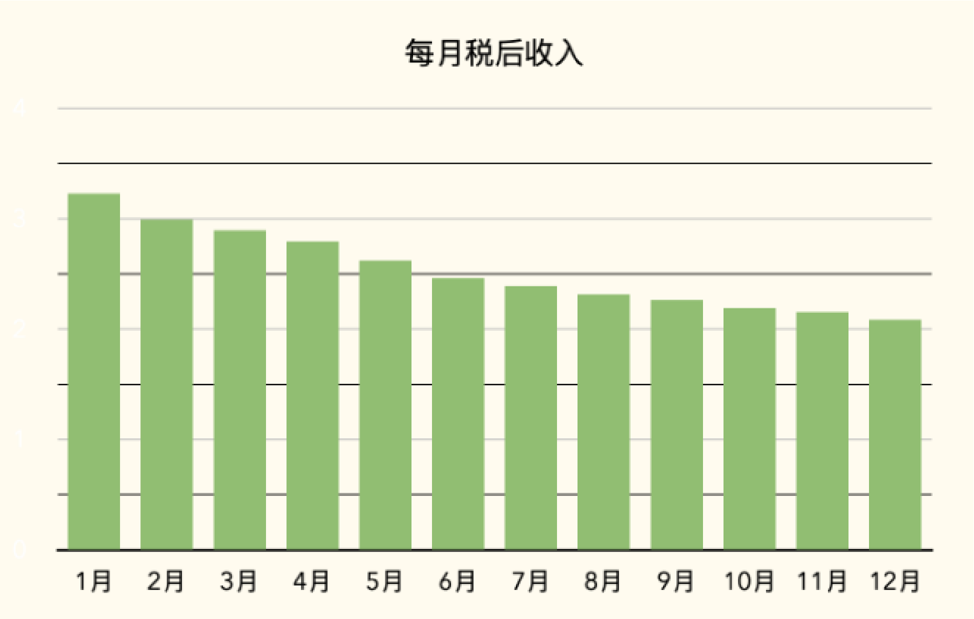

(Note: The Chinese characters "每月税后收入" in the figure translate to "Monthly After-tax Income," and the horizontal axis represents the months from January to December.)

(Note: The following two questions were included as a mental simulation exercise to enhance scenario immersion and to help participants mentally adapt to the hypothetical income scenario before consumption choice tasks.)

Q1. How satisfied are you with your continuously decreasing income?
(9-point Likert scale)
1 = Very dissatisfied … 9 = Very satisfied

Very dissatisfied								Very satisfied
1 ○	2 ○	3 ○	4 ○	5 ○	6 ○	7 ○	8 ○	9 ○

Q2. What impacts do you think decreasing income has on your life? *(Multiple choices allowed)*

☐ Reduced financial security and greater concern about future economic fluctuations

☐ Decreased planning for investment and financial management

☐ Reduced attention to personal health

☐ Fewer opportunities for leisure, travel, and social activities

☐ Lowering of living standards and reduced consumption of higher-quality products

☐ Regular saving for potential future needs

☐ No noticeable impact

☐ Other: __________

Module 3: Consumption Choice Tasks

Pre-Task Information:

No-Information Conditions:

(Note: The income-increase/no-information and income-decrease/no-information conditions serve as the reference conditions and provide no social information.)

Under the above scenario, assume that your stable income allows you to purchase each of the following products either in full or through monthly installments. In each case, please choose one option based on your true preferences.
(Images and prices are for reference only, but do not necessarily indicate the exact item

that you would buy. Your choice represents preferences for similar products at comparable price levels.)

Cheap-Salience Conditions:

(Note: These conditions combine either the hypothetical income-increase or income-decrease scenario with cheap-salience information.)

Under the above scenario, assume that your stable income allows you to purchase each of the following products either in full or through monthly installments. In each case, please choose one option based on your true preferences.
(Images and prices are for reference only, but do not necessarily indicate the exact item that you would buy. Your choice represents preferences for similar products at comparable price levels.)
The following product choice information (showing only the percentage of participants who selected the left-side item) reflects the choices made by other participants in a previous survey batch facing the same income scenario. This may serve as a reference for your decision-making.

Expensive-Salience Conditions:

(Note: These conditions combine either the hypothetical income-increase or income-decrease scenario with expensive-salience information.)

Under the above scenario, assume that your stable income allows you to purchase case, please choose one option based on your true preferences.
(Images and prices are for reference only, but do not necessarily indicate the exact item that you would buy. Your choice represents preferences for similar products at comparable price levels.)
The following product choice information (showing only the percentage of participants who selected the right-side item) reflects the choices made by other participants in in a previous survey batch facing the same income scenario. This may serve as a reference for your decision-making.

(Note: The phrase 'stable income' in the instructions above—applicable to all six experimental conditions—refers to the income trajectory assigned in each participant's respective scenario: consistently increasing in the three income-increase conditions and consistently decreasing in the three income-decrease conditions. The term denotes temporal consistency within the assigned scenario rather than equivalence of income levels across conditions.)

Attention Check (Common to All Groups):
To verify whether you answered seriously, please select "Very dissatisfied".
○ Very dissatisfied

○ Dissatisfied
○ Satisfied
○ Very satisfied
(If "Very dissatisfied" is not selected, the respondent is screened out.)

Product Choice Tasks:

(Note: Differences across groups were limited to:

No-information conditions: no information about previous participants' choices was displayed

Cheap-salience conditions: the percentage choosing the cheaper option was displayed

Expensive-salience conditions: the percentage choosing the more expensive option was displayed)

To illustrate the differences in social information across experimental groups, we use **Q3** (the first product-choice task) as an example. The information presented to participants in Q3 differed across the six groups as follows:

(Note on Imagery: To comply with copyright regulations, the original images from the experiment have been replaced with photos of identical angle, composition, and layout taken directly by the authors. These images were further processed using the AI tool Doubao for modification. Any logos or identifiable marks have been completely removed. Additionally, for copyright compliance, the specific brand names used in the original experiment (Luckin Coffee and Starbucks) have been replaced with generic categorical descriptions ("Mass-market Coffee" and "Premium International Coffee") in this appendix. The authors have reviewed and edited the outputs and take full responsibility for the content.)

No-Information Conditions (Income Increase and Income Decrease):

Q3. When passing by two different coffee shops, which coffee would you choose?

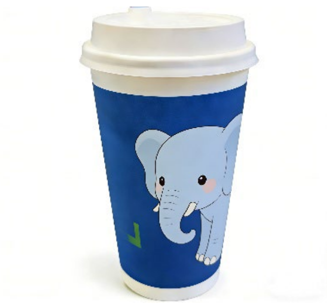

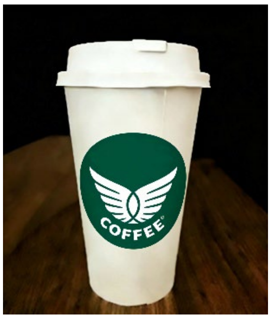

○ Mass-market Coffee (¥13.9)　　○ Premium International Coffee (¥33)

Income-Increase/Cheap-Salience Condition:

Q3. When passing by two different coffee shops, which coffee would you choose?

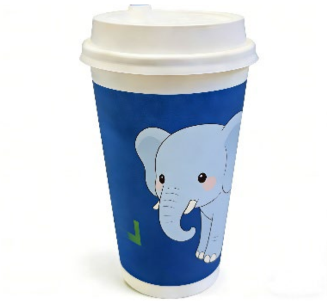

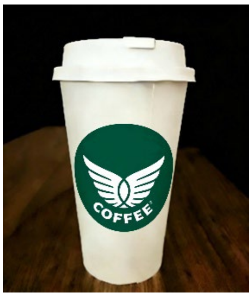

○ Mass-market Coffee (¥13.9) ○ Premium International Coffee (¥33)

○ 66.7% of people chose

Income-Decrease/Cheap-Salience Condition:

Q3. When passing by two different coffee shops, which coffee would you choose?

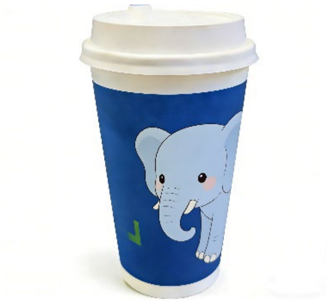

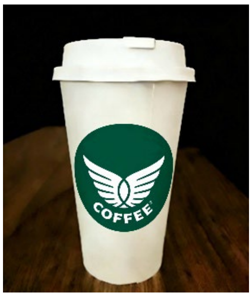

○ Mass-market Coffee (¥13.9) ○ Premium International Coffee (¥33)

○ 100% of people chose

Income-Increase/Expensive-Salience Condition:

Q3. When passing by two different coffee shops, which coffee would you choose?

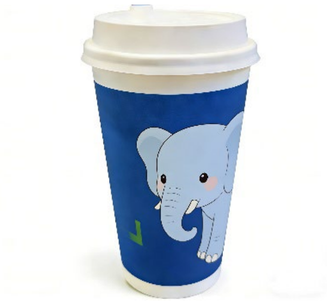

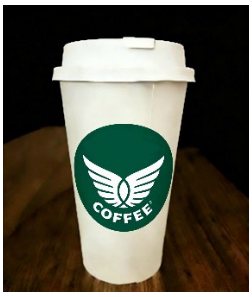

○ Mass-market Coffee (¥13.9) ○ Premium International Coffee (¥33)

○33.3% of people chose

Income-Decrease/Expensive-Salience Condition:

Q3. When passing by two different coffee shops, which coffee would you choose?

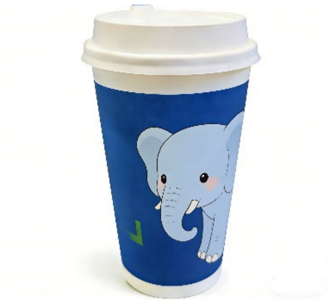

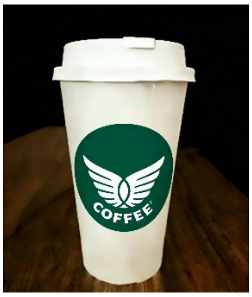

○ Mass-market Coffee (¥13.9)　　○ Premium International Coffee (¥33)

○0% of people chose

To display the full set of product choices, we present below the complete Product Choice Tasks for the income-decrease/cheap-salience condition:

(Note: The percentages of consumer choices shown differed by treatment group, depending on the income trajectories derived from previously collected choice data.)

Q3. When passing by two different coffee shops, which coffee would you choose?

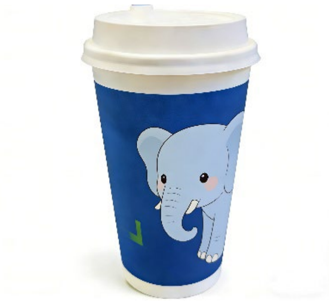

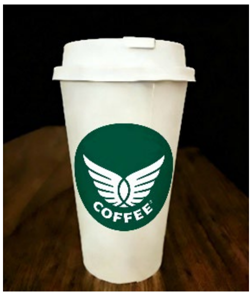

○ Mass-market Coffee (¥13.9)　　○ Premium International Coffee (¥33)

○ 100% of people chose

Q4. Which hat would you purchase?

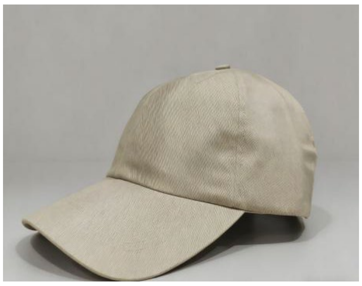

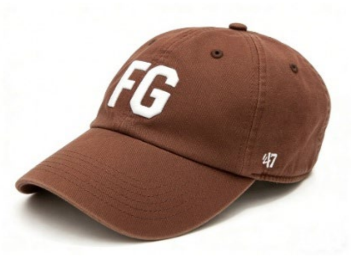

○ Standard baseball cap (¥37)　　○ Branded fashion baseball cap (¥227)

○ 86.7% of people chose

Q5. Which pair of shoes would you prefer to buy?

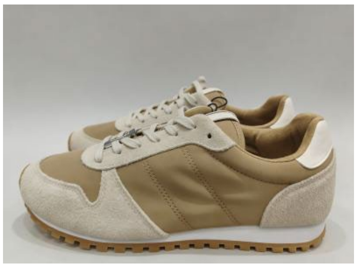

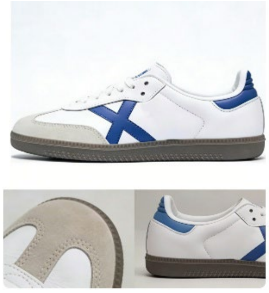

○ Standard shoes (¥99) ○ Premium brand shoes (¥799)

○ 76.7% of people chose

Q6. Which meal would you choose?

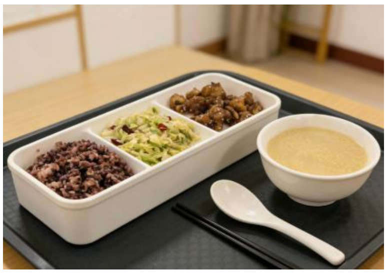

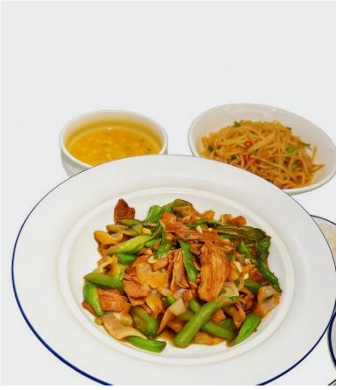

○ Boxed meal (¥15) ○ Stir-fried dish (¥27)

○ 56.7% of people chose

Q7. Which fruit would you choose?

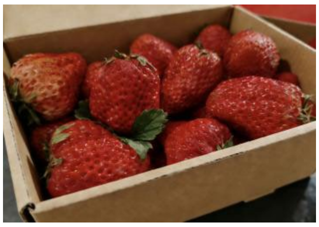

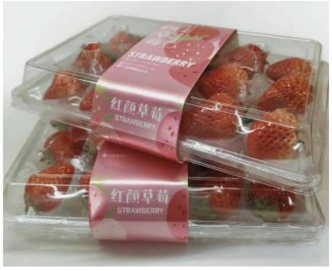

○ Bulk strawberries (25 pieces, ¥15)○ Selected premium strawberries (25 pieces, ¥45)

○ 83.3% of people chose

Q8. During leisure time, which sports equipment would you buy?

(Assume badminton; you may imagine another sport you are familiar with.)

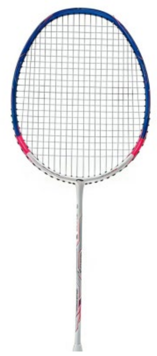 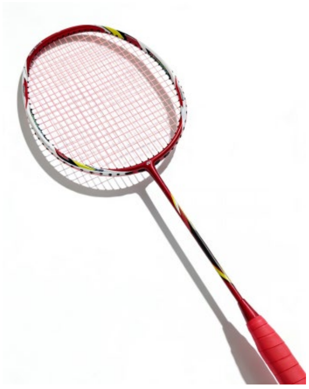

○ Basic badminton racket (¥114) ○ Full carbon badminton racket (¥560)

○ 86.7% of people chose

Q9. Which water bottle would you choose?

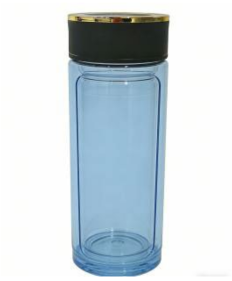 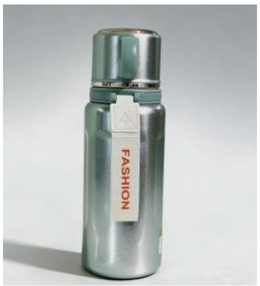

○ Practical water bottle (¥20) ○ Aesthetic, safe-material water bottle (¥82)

○ 46.7% of people chose

Q10. Which shampoo would you choose?

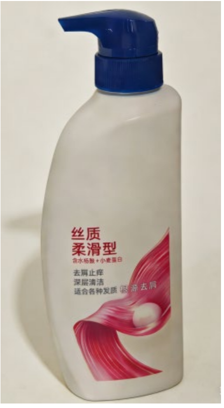

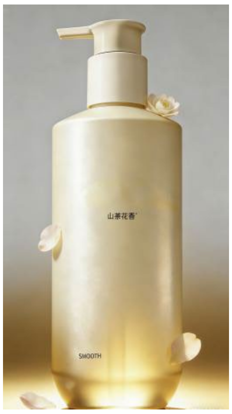

○ Basic shampoo (400 ml, ¥39) ○ Functional shampoo (400 ml, ¥120)

○ 63.3% of people chose

Q11. Which skincare product would you buy for yourself or close relatives/friends?

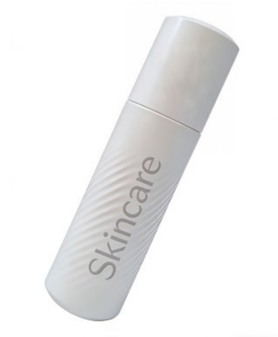

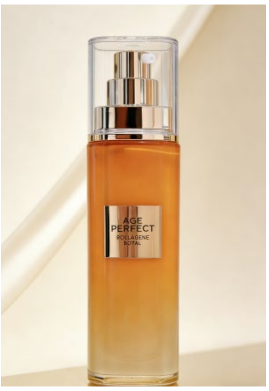

○ Basic skincare product (75 ml, ¥122) ○ Premium skincare product (75 ml, ¥530)
○ 70% of people chose

Q12. Which smartphone would you choose?

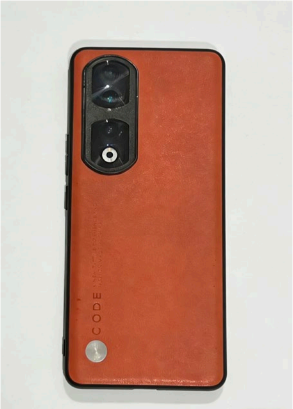

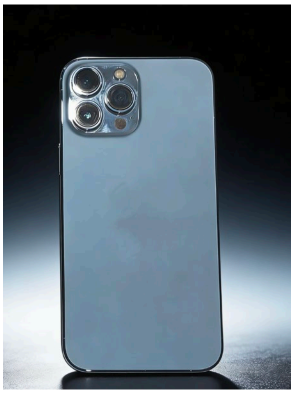

○ High-configuration domestic brand (¥4,775) ○ High-configuration international brand (¥7,199)
○ 86.7% of people chose

Module 4: Post-experiment Questions (Common to All Groups):

You have completed the main part of the survey. The following questions collect background information for research purposes only and will remain confidential.

Q13. Gender
○ Male
○ Female

Q14. Age
[Open-ended]

Q15. Highest level of education
○ Primary school or below
○ Junior high school
○ Senior high school / vocational school
○ Associate degree
○ Bachelor’s degree
○ Master’s degree
○ Doctoral degree

Q16. Marital status
○ Unmarried
○ Married
Q17. Do you have children?
○ None
○ One
○ Two
○ Three or more
Q18. City of primary residence
[Open-ended]
Q19. Area of residence
○ City
○ Town
○ Rural area
Q20. Current employment status *(Multiple choices allowed)*

☐ Student

☐ Unemployed

☐ Part-time employment

☐ Self-employed

☐ Employed by a company

☐ Employed by government or public institution

☐ Other: __________

Consistency Check
Q21. Under the income-decrease scenario described earlier, which smartphone would you choose?
(Please keep your answer consistent with your previous choice.)

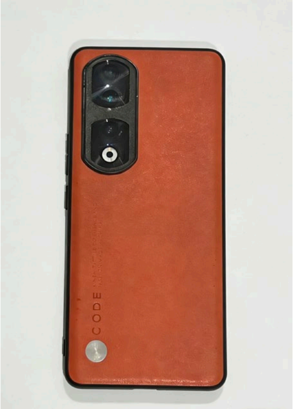

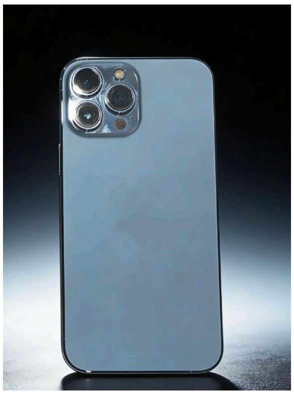

○ High-configuration domestic brand (¥4,775)

○ High-configuration international brand (¥7,199)

Q22. What are your main sources of monthly income in real life? *(Multiple choices allowed)*

☐ Financial support from family

☐ Scholarships or government subsidies

☐ Primary job income

☐ Secondary job income

☐ Investment or rental income

☐ Other: __________

Q23. How would you describe the asset level of your parents or guardians?

○ Wealthy
○ High-income
○ Middle-income
○ Low-income
○ Poor

Q24. Your approximate monthly after-tax income

○ Below 2000
○ 2001–4000
○ 4001–6000
○ 6001–8000
○ 8001–10000
○ 10001–15000
○ 15001– 20000
○ 20001–25000
○ 25001–30000
○ 30001–35000

○ 35001–40000
○ 40001–45000
○ 45001–50000
○ Above 50001

Q25. Your approximate monthly housing expenditure

○ Below 2000
○ 2001–4000
○ 4001–6000
○ 6001–8000
○ 8001–10000
○ 10001–15000
○ 15001–20000
○ 20001–25000
○ 25001–30000
○ 30001–35000
○ 35001–40000
○ 40001–45000
○ 45001–50000
○ Above 50001

Q26. What proportion of your income is spent on consumption each month?

○ 0–10%
○ 11–20%
○ 21–30%
○ 31–40%
○ 41–50%
○ 51–60%
○ 61–70%
○ 71–80%
○ 81–90%
○ 91–100%

Q27. Among your close family members, how many have adequate pension coverage?(Multiple choices allowed)

☐ None
☐ One
☐ Two
☐ Three
☐ Four
☐ Five
☐ Six
☐ Seven
☐ Eight
☐ Nine and above

Q28. What is your current trend in consumption expenditure?

○ Substantially decreasing

○ Slightly decreasing
○ No change
○ Slightly increasing
○ Substantially increasing

Q29. How do you view the economic outlook over the next few years?

○ Very pessimistic
○ Pessimistic
○ Slightly pessimistic
○ Neutral
○ Slightly optimistic
○ Optimistic
○ Very optimistic
